# Smart Hydrogels in Tissue Engineering and Regenerative Medicine

**DOI:** 10.3390/ma12203323

**Published:** 2019-10-12

**Authors:** Somasundar Mantha, Sangeeth Pillai, Parisa Khayambashi, Akshaya Upadhyay, Yuli Zhang, Owen Tao, Hieu M. Pham, Simon D. Tran

**Affiliations:** McGill Craniofacial Tissue Engineering and Stem Cells Laboratory, Faculty of Dentistry, McGill University, 3640 University Street, Montreal, QC H3A 0C7, Canada; somasundar.mantha@mail.mcgill.ca (S.M.); sangeeth.pillai@mail.mcgill.ca (S.P.); parisa.khayambashi@mcgill.ca (P.K.); akshaya.upadhyay@mail.mcgill.ca (A.U.); yuli.zhang@mail.mcgill.ca (Y.Z.); owen.tao@mail.mcgill.ca (O.T.); hieu.pham@mail.mcgill.ca (H.M.P.)

**Keywords:** regenerative medicine, hydrogels, tissue engineering, smart hydrogels

## Abstract

The field of regenerative medicine has tremendous potential for improved treatment outcomes and has been stimulated by advances made in bioengineering over the last few decades. The strategies of engineering tissues and assembling functional constructs that are capable of restoring, retaining, and revitalizing lost tissues and organs have impacted the whole spectrum of medicine and health care. Techniques to combine biomimetic materials, cells, and bioactive molecules play a decisive role in promoting the regeneration of damaged tissues or as therapeutic systems. Hydrogels have been used as one of the most common tissue engineering scaffolds over the past two decades due to their ability to maintain a distinct 3D structure, to provide mechanical support for the cells in the engineered tissues, and to simulate the native extracellular matrix. The high water content of hydrogels can provide an ideal environment for cell survival, and structure which mimics the native tissues. Hydrogel systems have been serving as a supportive matrix for cell immobilization and growth factor delivery. This review outlines a brief description of the properties, structure, synthesis and fabrication methods, applications, and future perspectives of smart hydrogels in tissue engineering.

## 1. Introduction

The lack of potential donors for organ transplantation has instigated researchers across the globe to find alternatives to combat the ever-increasing demand for organs. The past two decades have addressed this widespread issue by developing and integrating highly biocompatible yet sensitive materials for tissue regeneration. Hydrogels are a unique group of biocompatible 3D polymeric substances which can act as a scaffold and mimic the properties of various tissues in the body. The mechanism is by incorporating cells in their structure while eventually degrading themselves to leave behind only healthy tissue. They have gained widespread popularity in recent years based on their ability to retain high water content, maintain porous structure, and adapt by interchangeable sol-gel conditions. These structural qualities allow hydrogels to be used as tissue scaffolds in the body by promoting the influx of cell metabolites and the disposal of cell wastes through their pores.

With consistent efforts, researchers have strived to engineer these hydrogels by modifying their physical and chemical properties, referring to them as ‘smart’ or ‘intelligent’ hydrogels since they respond to external stimuli like temperature, pH, light, magnetic and electric fields, ionic strength, or enzymatic environment [1]. Smart hydrogels have shown great potential in non-invasive, remote-controlled therapies, including targeted drug delivery [2,3,4,5], regenerative medicine [6,7], tissue engineering [8,9,10,11], and implanting artificial organs [12,13]. However, these stimuli-based responses of hydrogels have shown limited applications and focus primarily on tissue specificity for success [14].

Constant research and advancement for improving the properties of these smart hydrogels by modifying their structural components and synthesizing methods have resulted in more innovative materials like supramolecular [15], micro-engineered [16], and nanofiber infused hydrogels [17]. This short review will summarize the evolution of hydrogels as scaffold materials in tissue engineering, their classification, properties, biomedical applications, and their current status in synthesis and fabrication techniques. We also aim to discuss the recent advances in smart hydrogels applications as well as their future perspectives in regenerative medicine.

### 1.1. Defining Biologically Active Scaffolds

Scaffolds are three-dimensional porous solid biomaterials which provide a physical surface for adsorption of biomolecules, and immobilization of proteins, growth factors and other biologically active biomaterials. These scaffolds/bioactive systems show great potential in tissue engineering and regenerative medicine in rendering bioactivity and specificity to the scaffold structure. According to Khandan et al., 2017, the polymer scaffolds function as space-filling agents, delivery vehicles for carrying bioactive peptides, and as three-dimensional structures. They aid in organizing cells and render the stimuli to direct the formation of the desired tissue. It is very crucial to find an acceptable material to deal with the crucial biological blueprint inherent to each application [18]. Scaffolds mainly serve as cellular matrices or as delivery vehicles for cellular contents, growth factors, and drugs in regenerative medicine; thus, the cellular material should be capable of colonizing the host cells to meet the requirements of repair and regeneration.

### 1.2. Need and Significance of Hydrogel-Based Scaffold Systems

Tissue engineering and regenerative medicine is a multidisciplinary field that aims at regenerating new biological tissues to replace and regenerate previously damaged structures. Most tissue engineering techniques combine the study of biology, biochemistry, clinical medicine, and materials science to achieve clinical applications.

Scaffold matrices play a very crucial role in the development of new tissue morphogenesis by guiding the growth of cells seeded within them or to help cells migrate from surrounding tissues by communicating with them. Most of the human cell types require adequate anchorage to support tissue regeneration, and thus the lack thereof may result in cell necrosis and defective tissues. “It is essential for the scaffolds to act as a substrate and have essential physical and chemical properties necessary to promote cell attachment, proliferation, differentiation, and migration” [19]. They deliver cells to the desired size in the patient’s body, provide a space for new tissue formation, and potentially control structural and functional integrity of the newly engineered tissue [20,21].

Over the years, various polymeric biomaterials have been developed by adding multiple functional groups in their molecular structure to control the physical, chemical, and biological characteristics of these scaffolds [22,23,24,25]. They are processed using materials both natural and synthetic in origin, derived from sources like algae, animals, micro-organisms [19] and synthetic biomaterials derived either from lactic acid, caprolactone, or glycoside monomers [26,27]. Though several scaffold matrices were introduced, which had sufficient qualities to provide necessary support and properties required for tissue growth, they had inadequate cell mimicking property and limited interaction with stromal cells which were crucial in promoting tissue regeneration.

An alternative, yet minimally invasive, approach developed to overcome the limitations of these polymeric scaffolds was the designing of hydrogel-based scaffolds for tissue engineering. Hydrogels are three-dimensional natural or synthetic polymeric networks in which the liquid component is water. Many elements of our body contain hydrogels in the form of the extracellular matrix, collagen, mucous, gelatin, cartilage, meniscus, epidermis, vitreous humour, and tendons. The hydrophilic and 3D structure of the hydrogels renders them the capability of holding substantial amounts of water or biological fluids [28]. The hydrogels are composed of dynamic crosslinking structure, which allows them to maintain the integrity of the hydrogel network and, therefore, they do not dissolve in high concentrations of water. The presence of high-water content in the hydrogel scaffold helps in the diffusion of nutrients. They also possess a significant degree of elasticity or flexibility similar to the native extracellular matrix (ECM) that provides structural and biochemical support to the surrounding cells, and it largely determines how a tissue appears and functions, making hydrogels a novel material for tissue engineering.

Hydrogels have been considered as the mainstay material of choice for tissue engineering applications since the past decade, according to Fan and Wang (2017), owing to their singular characteristics and gelatinous structure [29].

Thus, to make the use of hydrogels in clinical applications more relevant, it is essential to incorporate specific biological, clinical, and mechanical aspects, which are not only theoretical but also have a practical use. An appropriate scaffold must be capable of repairing body tissues with minimum requirements for cell growth, vascularization, proliferation, host-integration, and finally, the scaffold should be degraded naturally during or shortly after the healing process [30].

However, there are still several challenges in integrating these hydrogel systems to behave sequentially and rhythmically as per various physiological and pathological changes that occur continuously within the human body. Thus, a need to develop novel hydrogel scaffold systems to promote successful tissue regeneration for biomedical applications remains.

## 2. History of Hydrogels

The existence of hydrogels can be dated back to the 19th century, where they were colloidal gels made of inorganic salts. They were also one of the earliest biomaterials to be used inside the patient [18]; research on hydrogels as tissue engineering materials first began in the 20th century. First-generation hydrogel systems incorporated an extensive and diverse range of crosslinking procedures that involved chemical modification of monomer or polymer with an initiator. Initiators are chemical substances added to react with hydrogel polymers to create continuous cross-linking within their structure [21]. The method aimed to develop a biomaterial with a high affinity for water, excellent mechanical properties, and a simple mechanism of action [22]. The second generation of materials could respond to specific stimuli like variations in temperature, pH, or concentration of molecules in the solution; these targeted stimuli were targeted for specific events [22].

When scientists began trying to regenerate tissues and organs for transplantation, hydrogels—with their superior hydration properties—were considered as pioneers for the task. The initial focus was on improving the hydrophilic character of these gels to mimic the ECM structure and functions. Modifying the cross-linking combination of the chemical bonds and improving their physical interactions played a critical role in achieving this goal.

Despite advancements, some properties of hydrogels were short of expectations for tissue engineering—such as their biocompatibility, stiffness, and strength—thus, they required some modifications [31,32]. Several improvements were studied since then to address these drawbacks. For example, for higher biocompatibility, functional groups (e.g., Arg-Gly-Asp peptide) were introduced onto the surface of nanostructured scaffolds using chemical modification methods (e.g., plasma exposure) [33]. In another study, to reduce the toxicity caused by classical chemical cross-linking processes, argon micro-plasma was introduced as a neutral energy source for cross-linking in the fabrication of desired gelatin-graphene oxide (gel-GO) nanocomposite hydrogel scaffolds [34].

The progress and advancements in the science and applications of hydrogel systems have led researchers to develop ‘smart hydrogels,’ which are polymeric scaffolds with tunable properties and can be triggered by certain stimuli. Therefore, remarkable progress in hydrogel science has catapulted these classes of materials into a technological advancement, which opens up opportunities for further research in the coming decades.

## 3. Types of Hydrogels for Scaffold Design

Stimuli-responsive hydrogels (smart hydrogels)

Physical responsive hydrogels

❖ Temperature responsive hydrogels
❖ Photo/Light responsive hydrogels
❖ Electro- and magnetic responsive hydrogels

Chemical responsive hydrogels

❖ pH-responsive hydrogels
❖ Glucose responsive hydrogels
❖ Biological/biochemical-responsive hydrogels

### 3.1. Smart Hydrogels

Recently, considerable interest has been drawn to the so-called ‘smart hydrogels’, which are known to have the ability to respond to changes in their external environment. These polymers can exhibit dramatic changes in their swelling behavior, sol-gel transition, network structure, permeability, or mechanical strength in response to changes in the pH, ionic strength, or temperature, as an example. In comparison, the conventional hydrogels undergo only the swelling–deswelling process depending on the surrounding environment and availability of water.

Physical stimuli can include temperature, electric and magnetic fields, and light, while chemical stimuli include pH, ions, and specific molecular recognition events such as glucose. Some hydrogels can also respond to particular molecules like enzymes and antigens, which may elicit a biological or biochemical response. It is noted that pH and temperature-responsive hydrogels have been the most widely investigated among all the stimuli-sensitive hydrogels because these two factors have physiological significance inside the human body and can be easily controlled and applicable in vivo and in vitro conditions. Also, temperature-sensitive hydrogels are of particular interest for in situ forming ability.

Smart hydrogels have been used in many applications, divided into four broad areas, namely (i) controlled drug delivery (e.g., loading and release of bioactive molecules); (ii) biomedical applications (e.g., shape memory implantable devices, smart valves, coating in microfluidics and bio separation); (iii) biosensors (e.g., glucose sensors); and (iv) tissue engineering and regenerative medicine (e.g., injectable systems for the delivery of cells and growth factors, stimuli-responsive surfaces to control cell adhesion, or hydrogels that enable cell infiltration).

#### 3.1.1. Temperature Responsive Hydrogels

These are the most extensively studied class of hydrogel systems. Hydrogels are formed by polymer solutions having a lower critical solution temperature (LCST), which shrinks as the temperature increases above the LCST, thus displaying a nonlinear relationship with temperature, which is known as inverse temperature dependence. Thermo-responsive polymer hydrogels are characterized by the presence of hydrophobic groups in the monomers (e.g., methyl, ethyl, and propyl groups) and hydrophilic segments (e.g., amide and carboxyl). A large number of polysaccharides have been considered to be combined with these temperature-sensitive polymer hydrogels, including poly (ethylene glycol), chitosan, hyaluronic acid, chondroitin sulphate, alginate, cellulose, and dextran. This combination offers new possibilities to develop novel hydrogels [35,36,37].

#### 3.1.2. Photo-Light Responsive Hydrogels 

Hydrogels that respond to light are called light-responsive hydrogels, and they are very attractive for biomedical applications, primarily if visible light is employed. They are composed of a polymeric network and a photoreceptive moiety as the functional part. Stimulation by light induces changes in their physical and chemical properties. The optical signal is first captured by the photochromic molecules that convert the photoirradiation to a chemical signal through a photoreaction involving isomerization (cis-trans, open-close), cleavage, and dimerization. The latter signal is transferred to the functional part of the hydrogel and controls its properties. Since most of the studied light-responsive systems involve groups that react to UV light, it can limit the applicability in biomedical applications.

According to Qiu and Park (2012), photo-responsive hydrogels are of particular interest as carriers in drug delivery. The photoresponsive hydrogels have advantages since the light stimulus can be imposed instantly with accuracy. A photoresponsive hydrogel composed of cyclodextrin or azobenzene modified dextran was studied as a controlled delivery system of proteins and concluded that hydrogels that are responsive to specific molecules are of particular interest to be used as drug delivery systems. However, the response time of these hydrogel systems is too slow. Thus, fast-acting hydrogels are necessary and require significant improvements in the hydrogel properties [38].

#### 3.1.3. Electro and Magnetic Responsive Hydrogels 

Electric and magnetic field responsive hydrogels change their properties in response to small changes in electric current stimuli or external fields, respectively. These systems have been investigated as a type of hydrogel that swells, shrinks, or bends. Typically, these hydrogels have been studied in the form of polyelectrolyte hydrogels (systems that contain a high concentration of ionizable).

Synthetic polymers that have been used to develop electroactive hydrogels include polyvinyl alcohol, acrylic acid/vinyl sulfonic acid, or sulfonated polystyrene, for example. Naturally-occurring polymers are also commonly employed to prepare electro- and magnetic-responsive materials. For example, alginate, hyaluronic acid, and chitosan have been blended with synthetic polymers to make such hydrogels [39,40].

#### 3.1.4. Chemical Responsive Hydrogels (pH-Responsive Hydrogels)

Classical pH-sensitive polymers used in hydrogel preparation are poly(acrylic acid) (PAA), polyacrylamide (PAAm), poly(methacrylic acid) (PMAA), poly(diethylaminoethyl methacrylate) (PDEAEMA), and poly(dimethylaminoethyl methacrylate) (PDMAEMA), and their copolymers.

These polymers contain hydrophobic moieties and swell in water, depending upon the pH of the external environment. On the other side, various natural polymers—such as chitosan (anionic), alginate (cationic), albumin, and gelatin—also show pH-responsive swelling behaviour. Due to the pH-sensitive character of these polysaccharides, a combination of these polymers with a thermo-responsive material can produce dual-stimuli responsive polymeric gels to be used as delivery vehicles that respond to localized conditions of pH and temperature in the human body. pH-responsive gels show great promise in tissue engineering applications, especially in the delivery and incorporation of bioactive agents. These classes of hydrogels have been most often used to develop for controlled drug release for oral administration and have also been designed for the delivery of insulin in clinical practice. A study establishing the application of pH-responsive hydrogels for protein drug delivery was conducted using a chitosan-based derivative, N, O-carboxymethyl chitosan(NOCC) along with alginate hydrocolloid which was blended to genipin, a fruit extract, to form a polymeric carrier which exhibited favourable results in achieving site-specific protein-based drug delivery [41].

#### 3.1.5. Glucose Responsive Hydrogels

Glucose-sensitive hydrogels have been investigated since they are exceptional candidates for developing insulin delivery systems that can act as an artificial pancreas to administer an exact amount of insulin in response to blood glucose concentration. The need for better control of blood glucose levels in diabetic patients has led to increased research in this field. The most general approach to the development of self-regulated insulin release systems that respond to blood glucose concentration involves the immobilization of the enzymes glucose oxidase (GOD) and catalase into a pH-responsive hydrogel enclosing a saturated solution of insulin. This change in concentration gradient promotes the swelling of the hydrogel and increase of network mesh size, resulting in a facilitated diffusion of the previously entrapped insulin out of the matrix. Upon the release of insulin, the sugar levels drop, resulting in a pH increase that stops the release of further insulin [42,43,44,45,46].

#### 3.1.6. Biological-/Biochemical-Responsive Hydrogels 

Bio-responsive hydrogels are systems that contain biological instructive functionalities/moieties and have been designed to interact with the biological environment. The in vivo applicability of bioresponsive hydrogels makes them particularly compelling. Concepts of interest include the use of biomolecules—typically peptides sequences cleavable under the action of specific enzymes that act as the crosslinking of the hydrogel system [47]. The catalytic activity of the enzyme on the substrate can lead to changes in swelling properties of the gel. Some examples are the families of enzyme matrix metalloproteinases (MMPs), which have the property to breakdown ECM molecules and are extensively involved in tissue development and remodelling [48,49,50]. Since enzymes are highly selective in their reactivity and are vital components in various biological pathways, materials can be programmed to respond to a specific enzyme by incorporation of the specific substrate. (Overview of the types of hydrogels and their applications are highlighted in Table 1).

## 4. Methods of Preparation of Hydrogels 

The primary structural component of a hydrogel is largely based on these two parameters: the amount of water the hydrogel is expected to absorb, and the method of binding the polymer chains within the gel network. Hydrophilic polymers can absorb different amounts of water depending upon the density of the hydrophilic groups present on the polymer. Water absorption for these polymers can range from a fraction to several thousand times their weight. The swelling characteristics of a hydrogel are critical parameters in their use in diverse applications because the equilibrium swelling ratio to weight ratio of swollen hydrogel over the dry hydrogel influences the solute diffusion coefficient, surface wettability and mobility, and the optical and mechanical properties of the hydrogel. The physical properties of swollen hydrogels are regulated by the molecular weight (MW) of the polymer, charges on the polymers, density of the cross-linking (covalently bonded networks), and physical associations. Each of these conditions helps define the relative amount of bonding between polymer chains. These properties are essential in the protection of encapsulated biomolecules from mechanical deformation at the transplantation site.

### 4.1. Free Radical Polymerization

**Figure 1:**
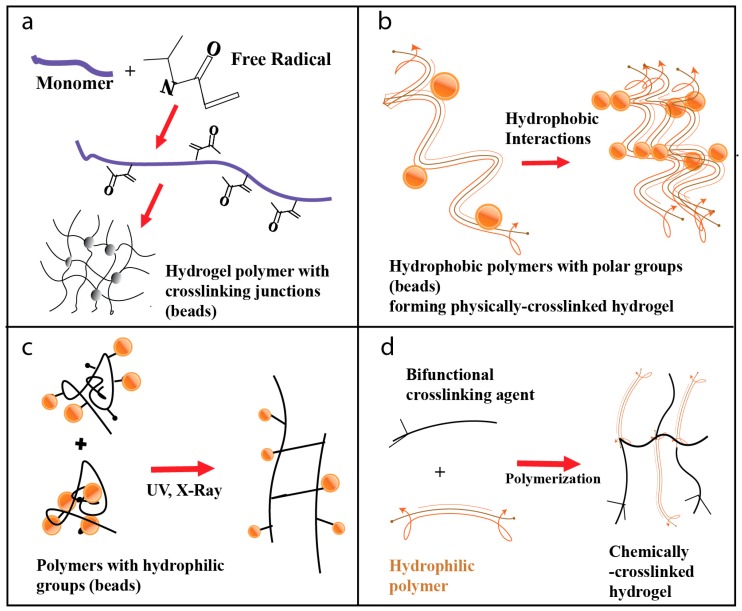
Synthesis of hydrogels in (**a**) free radical polymerization, (**b**) physical cross-linking, (**c**) irradiation cross-linking, and (**d**) chemical cross-linking.

Conventional free radical polymerization is the most preferred and versatile technique for the preparation of hydrogels based on some monomers such as acrylates and amides [51]. This method is used for the development of polymer-based hydrogels. It is a type of chain-growth polymerization and aids in the synthesis of nanocomposite hydrogels. In this method, the polymer is formed by the sequential addition of free radical groups, which act as templates for structural build-up (Figure 1a). This method follows the typical free radical polymerization steps in the sequence of initiation, propagation, chain transfer, and termination [52]. There are four methods of radical polymerization: bulk polymerization, solution polymerization, suspension polymerization, and emulsion polymerization. When large quantities of hydrogel preparation are desired, this method is employed.

### 4.2. Physical Crosslinking of Hydrogel Polymeric Precursors

This method involves the crosslinking of polymers through physical interactions. Physical cross-linking induces physical cross-links between polymer chains through intermolecular force. They are synthesized by ionic interaction, crystallization, protein interaction, and hydrogen bonding. Under physiological pH and standard room temperature, ion interaction methods are used to create hydrogel cross-links. This method does not require the presence of ionic groups, unlike other methods. Cross-linking via hydrophobic interactions results in a polymeric structure that stimulates uptake of water and swelling of the hydrogel that is essential for the formation of the hydrogel structure (Figure 1b). Hydrophobic interactions are crucial in preparing polysaccharide hydrogels, such as chitosan and dextran hydrogels. Protein interactions are usually made using block copolymers containing aqueous solutions in water, which undergo a transformation from sol to gel under physiological conditions [28].

### 4.3. Irradiation Crosslinking of Hydrogel Polymeric Precursors

This method has been proposed for the production of hydrogels and for obtaining a biocompatible matrix that can incorporate and release active ingredients in a controlled fashion. Ionizing radiation can be applied for the synthesis and modification of the internal structure and physicochemical properties of the polymers. Ionizing radiation has the property to modify the physical, chemical, and biological properties of the irradiated materials. Ionizing radiation techniques, in combination with the simultaneous sterilization process, are a very effective method for the synthesis of hydrogels [53].

High energy electromagnetic radiation, such as X-rays; or high energy particle beams, such as electron beam or gamma rays, are used to ionize simple molecules in air or water medium since they have high energy. Different sources of radiation are applied to the polymerization process. Gamma rays, X-rays, accelerated electrons, and ion beams initiate polymerization reactions by the formation of energetic radicals. During the irradiation of a polymer solution, reactive sites are generated along the polymer strands. The combination of these radicals will then lead to the formation of many cross-links in the polymeric structure [54]. This method is simple, and the extent of crosslinking can be controlled by varying the dosage of irradiation (Figure 1c). This procedure of fabrication has an advantage over other techniques of fabrication because no catalysts or additives are required to initiate the reaction. Sterilization and modification of polymeric structures can also be achieved simultaneously. The properties of the materials can be tuned and intentionally modified by varying different radiation parameters such as irradiation dose, frequency, temperature, and pressure. A wide range of hydrogels for many biomedical applications is synthesized using this technique due to the advantages it offers.

### 4.4. Chemical Crosslinking of Hydrogel Polymeric Precursors

This method is one of the significant and more commonly employed methods of hydrogel fabrication. Crosslinking hydrogels are chemically synthesized by chain-growth polymerization or addition, by condensation polymerization reaction, or by sequential addition of cross-linking agents. Hydrogel polymers are prepared by adding a bifunctional crosslinking agent to a dilute solution of a hydrophilic polymer having suitable functionality to react with the agent (Figure 1d). This method can be used for the preparation of both natural and synthetic polymer hydrogels. For example, albumin- and gelatin-based hydrogels were developed using di-aldehyde or formaldehyde as crosslinking agents [55].

## 5. Fabrication of Hydrogel Scaffolds in Tissue Engineering

A variety of fabrication techniques are available for the production of hydrogel-based scaffold matrices. These methods have been used for several years to help researchers integrate new generation porous scaffolds. However, not all of them are used today due to the evolution of more advanced and precise methods such as 3D printing and additive fabrication. This section will highlight some key hydrogel fabrication technologies in tissue engineering.

### 5.1. Emulsification

This simplified, yet effective, method of gel particle formation is a commonly used technique in constructing Nano- or micro-sized spherical gels used in tissue engineering. The working principle requires the mechanical mixing of a multi-phased solution with a hydrophobic phase like oil to form minute hydrogel droplets. Different sized droplets can be obtained by merely modifying the hydrogel precursor’s viscosity, the intensity of mixing, and regulated use of surface acting agents to limit hydrogel’s surface tension and prevent their agglomeration. A notable advantage of this method is their ability to formulate cell-laden hydrogel scaffolds by simply adding cell types to phase mixture, which in turn gets encapsulated within the gels. Nevertheless, this method only produces spherical gel structures and hence has limited use in various biomedical applications [56].

### 5.2. Freeze-Drying (Lyophilization) 

**Figure 2:**
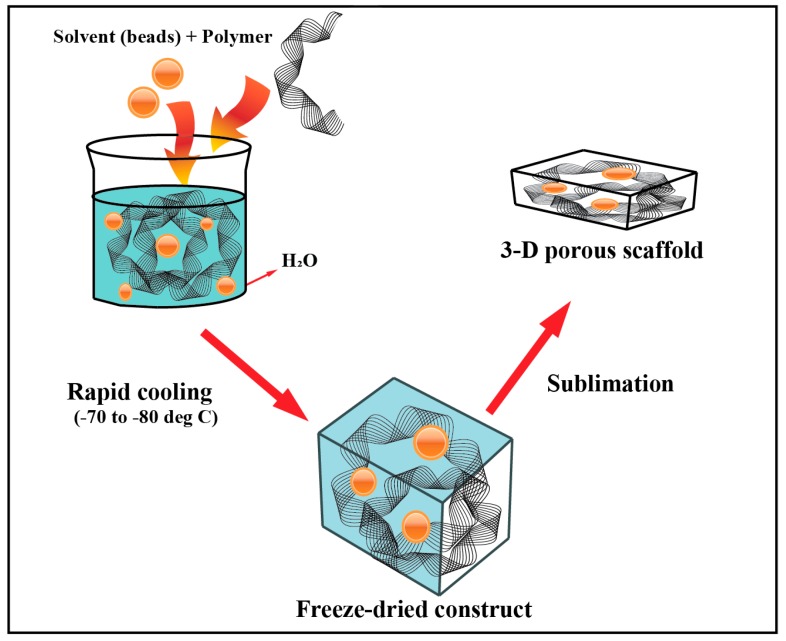
Freeze-drying (lyophilization) technique.

In this method, a polymeric substance and a solvent are added to water to form an emulsion. This solution undergoes rapid cooling to a temperature ranging from −70 °C to −80 °C, which leads to thermal instability within their structure. Furthermore, this solidified matter under partial vacuum is subjected to sublimation allowing the solvent to evaporate and eventually form porous voids in the freeze-dried structure building the scaffolds [33,56,57] (Figure 2). Lyophilization aids in controlling the pore size of scaffolds and hence, the permeability of these matrices. Numerous types of research conducted on this fabrication method demonstrated the ability to maintain a porous network. For example, Autissier et al. developed a highly-porous scaffold system, with polysaccharide as the key polymer, and subjected it to rapid cooling and sublimation. He concluded that this method could regulate both pore diameter and porosity within the scaffolds [58].

### 5.3. Porogen Leaching

**Figure 3:**
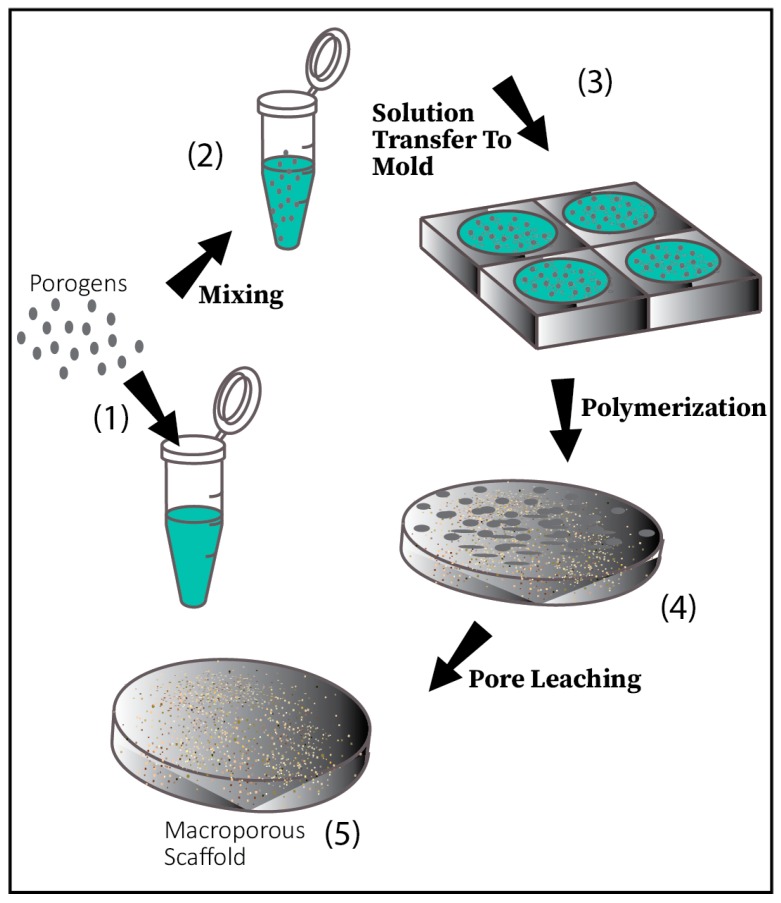
Porogen leaching technique.

This method is one of the most commonly used, convenient techniques to build hydrogel-based scaffolds. Here, salt particles are selected based on the desired pore size by grounding them adequately. These particles are transferred to and mixed with a solvent, which is synthesized by the use of biocompatible organic polymers. Once the desired shape is formed, the mold is subjected to freeze-drying, causing the solvent to evaporate and subsequent leaching of the trapped salt particles within the network, which creates the porosity necessary for tissue engineering applications [57,59] (Figure 3). However, one concern with this method is the presence of residual salt within the network forming defective pore structures [56].

### 5.4. Gas Foaming

Gas foaming utilizes the gaseous effervescence to devise highly porous interconnected scaffold networks. This technique uses a solvent-free approach and uses salts such as ammonium and sodium bicarbonate as gas-forming agents. In this technique, the moulded biodegradable polymer is condensed with the salts first, and then by either chemical or physical means, gas bubbles are generated within the mold, which leads to decreased solubility when the polymer is saturated. Consequently, pressure builds up, causing gas nucleation and thus the formation of interconnecting pores [60]. Studies have shown that this method has been utilized to develop scaffolds with macroporous structures and an ability to form uniformly sized pores ranging from 100 to 200 µm [61]. The use of citric acid and other acidic salts to promote gas nucleation has also been studied [62].

### 5.5. Electrospinning 

Electrospinning is one of the oldest and relatively economical methods used in fabricating fibres of submicron sizes with good mesh formation [63]. It utilizes a syringe pump, a high voltage direct current supply, and an earthed rotating collector. Firstly, a current is applied to the syringe tube, which is filled with the polymeric material; this leads to electric repulsion within the polymer solution, which jets the polymer out of the nozzle tip as thin filamentous strands. These fibres are collected by the rotating target collector, depending on the requirement of scaffold properties. The size achieved by this technique is usually smaller compared to other methods. This gives the scaffold superior cell interaction with good proliferation, attachment, and differentiation [64,65].

### 5.6. 3D Printing 

Conventional techniques for hydrogel fabrication are nowadays replaced by the use of state-of-the-art, sophisticated 3D printing technologies, which not only produces the desired size and shapes but even more precisely structurally and functionally versatile scaffold systems. 3D printing provides unprecedented control for constructing biological structures for transplantation and accurately control the geometrical parameters and cell distribution within the scaffold to mimic the transplanted organ with their 3D design [66]. The technique relies on the computer-aided design of the tissue to be transplanted and subjected to rapid prototyping. leading to the formation of layer by layer scaffold network by ink droplets, forming a complex tissue architecture for implantation [56]. However, inkjet/3D printing has limitations when it comes to resolution, choice of materials, and their cost of fabrication [59]. More extensive studies are required to improve inks designed with biological components for use in tissue engineering.

### 5.7. Photolithography

This top-down engineering method has been used widely in recent years to synthesize polymeric 3D scaffolds based on selective illumination techniques [57]. The construction follows a two-step method. Firstly, the photoresponsive polymer is masked with desired shapes and sizes kept over the substrate and subjected to UV light, which allows photopolymerization within the exposed areas. Then, the unreacted, unexposed, polymeric substrate is eliminated with a solvent to create the patterned 3D scaffolds [67]. Photolithography due to its highly specific fabrication technique can be utilized to create a 3D pattern in larger surface areas and maintain excellent architecture and alignment of the desired pattern. Several other techniques following this principle are reviewed to increase the ease of developing better hydrogel-based scaffolds. For example, the use of blue light to form a cross-linkable photopolymer has been studied for its use in tissue engineering [68]. Research on the use of lasers for improving cross-linking properties is under investigation [69].

### 5.8. Sol-Gel Technique 

This a chemical fabrication technique that involves the formation of a sol or a colloid solution, which involves both a liquid and solid phase within them, making them diphasic. The structure of the gel formed is mainly attributed to their morphological variances in the biphasic system (solid and liquid phase) used in hydrogel fabrication. The significant advantage of this method is the superior purity and uniformly laid nanostructure achieved at low temperatures.

The scaffold is usually fabricated by the dissolution of organic or inorganic metal compounds in a solvent and subjected to multiple cycles of hydrolysis and polymerization reactions to form a colloidal suspension or a sol structure. Furthermore, this structure is cast into a mould to achieve the gel structure, which, after subsequent dying and heat application, delivers nanoparticle-based hydrogels. Sol-gel technique has been used frequently nowadays due to its enhanced homogeneity and ability to undergo transitions at relatively low temperatures. They also effectively regulate the particle size and network morphology, which makes it a suitable technique to construct a highly specific type of hydrogels for complicated tissue replacement. The hydrogels formed using the sol-gel transition allows the addition of doping substances within the polymer network without causing chemical imbalance and physical deformities to the added agents. These advantages allow their use of different types of sensors used in biomedical tissue engineering and also in delayed drug delivery [70].

## 6. Properties and Structure

### 6.1. Mechanical Properties

Hydrogels are made of polymeric networks that might have strong chemical crosslinks or weaker physical associations. A gel is an intermediate state between liquid and solid. Due to the inhomogeneity in the structure with dangling chains and loops, mechanical properties are weak. Modulus of elasticity can range from kilopascals (kPa) to megapascals (MPa) [71]. Depending on the type of tissue to be regenerated, the requirement of the strength of the gel can vary. The mechanical properties of these gels have been evaluated by shear or compression- not by stretching because of poor deformability [72,73]. Thus, there have been many efforts to prepare new types of gels capable of large deformation. The first gel was the slide-ring (SR) gel discovered in 2001 by Okumura and Ito [74]. This gel was made by crosslinking polyrotaxane; the polyrotaxane consists of poly(ethylene glycol) (PEG) threaded through a ring molecule of cyclodextrin (CD). As there is no covalent bond between the (CD) molecule and the axis polymer—i.e., PEG—the crosslinks can slide along the axial chain, thus enabling deformation without destruction of bonds. The SR gels have unique mechanical and swelling properties, as extensively discussed by Ito [75]. Likewise, there has been the development of nanocomposite gels by Haraguchi and Takehisa [76], double network gels by Gong et al., 2003 [77], tetra-PEG gel by Sakai et al., 2008 [78]; Shibayama et al., 2012 [79] have extensively reviewed these.

Out of all mechanical properties, Young’s modulus has been most widely assessed, according to Peppas et al., 2000, [71]. Standard conventional methods analyze dynamic mechanical behaviour under tension or test viscoelastic properties under shear strain. For example, for uniaxial tensile tests, dumbbell-shaped hydrogel samples are prepared and subjected to varying loads under constant extension rates. Young’s modulus can be obtained by the slope of the graph by plotting the stress applied versus the strain produced [80]. Another method is of Atomic force microscopy. In this, an indentation is created by an atomic force microscopy (AFM) tip. The Young’s modulus is calculated by plotting a curve between force and indentation. Here the force is calculated by deflection and spring constant of the cantilever [81].

The tunability of these mechanical properties is crucial in directing the development of cells in their migration, proliferation, as well as differentiation [82]. Mathew and Denis have shown effect of stiffness of the gel on orientation and thus migration of cells during wound healing [83]. As a result, there has been a lot of work in mechanoresponsive hydrogels whose properties can be tuned just like natural tissues. Xiao et al. developed a cross-polymerized gel which released drug in response to an applied force [84].

### 6.2. Rheology 

Rheology has to do with the flowability of the gel. The viscosity is temperature dependent, following scaling laws at low temperatures and flowing more freely at higher ones. The rheological property assessed, corresponding to material stiffness qualitatively, is called shear storage modulus (G’). Likewise, loss modulus (G”) is assessed, which corresponds to material liquid flow properties. Measurement of these properties gives an insight into the changes in mechanical properties under shear. Improvement in this assessment has been suggested by many authors using other aids such as confocal rheometric assembly and particle tracking velocimetry [85]. This is because the conventional methods were based on three common assumptions that are: (1) no-slippage on the boundary; (2) homogeneous velocity profile of the bulk during shear; (3) relaxation upon shear cessation occurring without the emergence of macroscopic motion due to shear history [86]. Guilia Fanesi et al. (2018) demonstrated the potentiality of using rheology and low-frequency magnetic resonance imaging for the characterization of hydrogels [87].

The thixotropic behaviour of hydrogels, which is due to shear dependent fracturing, makes them indispensable to be used as injectables. Injectable hydrogels are gaining a lot of importance as they give a comfortable environment to the cells as they experience less shear and have more nutrients around them during the injection. Also, the gels can be moulded and take up the shape of the cavity or space where the tissue is expected to regenerate [88]. They can also be used for prolonging drug delivery over a period of time to enable the sustained presence of a drug [89].

### 6.3. Degradation Profile

All hydrogels eventually have to undergo degradation as they act as a supporting media for the tissues to grow. Degradation of hydrogels has to be precisely synchronized with the proliferation of cells in tissue regeneration, as the cells need space to grow. An early degradation can lead to cell death and the ultimate failure of regenerative tissue due to a lack of cushioning as well as nutrients. On the other hand, if it is delayed then it increases the chances of an immunogenic reaction and also suffocation of the growing cells. Additionally, for controlled release of drugs too, their degradation has to be closely monitored [90]. A lot of studies have been done to visualizing this phenomenon as done by Mohammad et al. (2018) using MRI [91]. There have also been efforts to study the non-specific degradation mechanism by Li et al. (2014) [92]. Such efforts have led to better understand and plan for control of the process. This control can be achieved by modifying the composition and structure of gel [93]. Laser-controlled degradation is also seen as an emerging trend that efficiently monitors the process and thus seems very promising [94].

### 6.4. Surface Properties

The surface of hydrogels varies greatly from rough to smooth, or crystalline to disordered matrices. These organizational and structural properties have been seen to significantly influence the fate of the cells in the tissue regeneration process, as the extracellular matrix in the natural tissues. Various methods to determine these properties are scanning electron microscopy (SEM), Fourier transforms infrared spectroscopy (FTIR), scanning tunnelling microscopy (STM), and AFM. Recent studies have integrated topographical changes to direct the development of cells/tissues according to their requirements. Unadkat et al. introduced random surface features with 2,176 different topography and grew human mesenchymal stromal cells on them to study the effect of topographies on osteogenic differentiation and proliferation of the cells [95].

### 6.5. Hydrogels with Enhanced Physical and Mechanical Properties

Hydrogels remained a pioneer group of materials in tissue engineering since the past decade or so. However, their low stress-bearing ability and reduced thermal conductivity have always been an area of concern which has limited their use in several tissue engineering applications. This limitation has urged the researchers to find new alternate hydrogel systems with better loading properties and heat resistance. DN (double-network) hydrogels are one such entity in hydrogel family which were developed to enhance their mechanical properties to find use in more application in tissue engineering.

The basic formation of DN hydrogels includes two steps. Firstly, 2-acrylamide-2-methyl propane sulfonic acid (AMPS) along with crosslinking agent N.N’-methylenebisacrylamide (MBAA) is used with 2-oxoglutaric acid as an initiator. The second network made of acrylamide (AAm) again containing an MBAA and initiator is merged into the first network, which finally forms the double network hydrogels PAMPS/PAAm [96].This method of DN hydrogel preparations is continuously investigated further because of improving the mechanical properties, including tensile strength and toughness of the material, by combining it with materials like gelatin, cellulose, and others among many to achieve more desirable properties suitable for several biomedical applications.

A study conducted by Xing et al. (2019) demonstrated the use of hydroxylated boron nitride nanosheets (0H-BNNS), which is shown to improve the thermal conductivity of the hydrogels. When cartilaginous tissues are subjected to long-time stress and friction, they tend to generate more heat, and with the introduction of OH-BNNS, these engineered tissues help improve the thermal conductivity and heat dissipation. Such modification improves the properties of DN hydrogels opening a new realm of possibilities in mimicking and simulating natural body tissues and structures [97].

Chemically–chemically crosslinked and physically–chemically crosslinked methods are the two conventional techniques used in the synthesis of DN hydrogels. However, the chemical methods are more often used since they can be modified quite easily and also can be used in combination with multiple gel systems. Although there needs to be more research conducted in developing DN hydrogels for better mechanical properties, withstanding high loading unloading stresses, it is evident from the current literature review that DN hydrogels provide superior load-bearing capacities, toughness, and thermal characteristics when compared to conventional hydrogels systems [98].

## 7. Characterization of Hydrogels 

The parameters based on which the hydrogels are characterized are biocompatibility, biodegradability, bio adhesion, dielectric relaxation, and mass transport properties [99].

### 7.1. Biocompatibility

The main concern to successfully use biosensors, drug release devices or neuronal prosthesis in vivo would be undesirable interactions between the host and the indwelling device [99]. One way to avoid this issue is to molecularly engineer the hydrogels and make them favorable to the immune system of the host [99]. Certain materials, such as PEG, are shown to resist protein adsorption, which is a likely precursor to the immune response [99]. In other types of films, such as the poly(HEMA)-based hydrogels, the water content and elastic moduli are designed to be as similar as the body tissues hence they would be compatible [99]. This parameter was evaluated using hyaluronic acid (HA)-based hydrogen film [100]. Using scanning electron microscopy (SEM), the structure of the gel was analyzed before and after hydration, showing a change from featureless to a porous microstructure after swelling [100]. The morphology of the hydrogels also gives information about changes in their transparency using digital microscopes [101].

### 7.2. Thermal Behaviour

To characterize the reactions of the gel to thermal changes, differential scanning calorimetry can be used [82]. For this analysis, a drying cycle needs to be introduced in advance to avoid the broad peaks of water in hydrogel samples, and the heating speed would be about 10 °C/min with subsequent analysis from 0 to 200 °C [101].

### 7.3. Responsiveness to pH 

One of the applications of hydrogels is in wound dressings as the pH of the wound changes overtime and hydrogels happen to react to the change in pH [101]. The swelling characteristics of the hydrogels were analyzed based on the common pH of the wounds (5–9) [101]. Studies show that there may be a relationship between the amount of GAN (the acid welling n hydrogel, which crosslinks with hydroxyl groups) and the swelling capacity of gels. The higher GAN levels in the composition are associated with comparable swelling in alkaline and neutral pH and lower water absorption in acidic environments [101].

### 7.4. Swelling Studies

Swelling is measured and presented as a percentage value. Hydrogel films as small as 1 cm^2^ would be weighed before and after being swollen in water for five hours at room temperature. The films would get removed and dried with filter paper at regular time intervals to avoid the surface water interfering with the osmosis of water and its rate [83]. After five hours, the gel piece would be weighed again, and based on the following formula, swelling percentage would be estimated:

% Swelling = (Final mass − Initial Mass)/Initial Mass(1)

In other studies, methylene blue loading and release can be used to evaluate the swelling properties of the gel. The 1 cm^2^ gel pieces would be loaded with methylene blue by immersing them (dry) in the dye for a period of time between 1 h to 24 h. Knowing the concentration of the dye, loading characteristics would be evaluated by measuring its absorbance before and after the presence of the hydrogel films [101]. The absorbance values would be obtained using a UV–vis plate reader at the wavelength of 664 nm.

### 7.5. Crosslinking Degree

To evaluate the crosslinking properties of the polymer films, attenuated total reflectance (ATR)–Fourier transform infrared (FTIR) spectroscopy can be used [101]. The IR spectra can be recorded at room temperature based on multiple scans [83]. While analyzing the interactions taking place between the hydrogel material and GAN are compared with the non-covalent interactions in the pure hydrogel material. The anhydride groups, new ester groups, and broadened GAN peaks are the significant changes that the IR spectrum reveals [101].

### 7.6. Porosity and Permeation

There exists a network of pores that vary in size, distribution, and interconnections and would make up the matrix of the hydrogel film and the resulting structure in it [102]. The parameter ‘tortuosity’ defines all three characteristics of the pores and the composition of the gel, along with its crosslink density, influences the pre volume and tortuosity [102].

### 7.7. In Vitro Microbiological Assessment 

Biofilm materials may serve as reservoirs for pathogenic infections in the host’s body as bacteria tend to adhere to the surface of the hydrogels and usually show resistance to the host’s immune system [101]. The resistance of hydrogels can be evaluated in vitro by equipping them with anti-adherent properties, mainly provided with an inoculum of the bacteria over incubation periods to prevent infections [101].

## 8. Application of Smart Hydrogels for Tissue Engineering

The field of tissue engineering uses the principles of engineering and life sciences intending to maintain and revitalize the functions of tissues and organs (Khandan et al., 2017) [18]. Smart biomaterials such as hydrogels are generally employed in health care and biomedical sciences because of their similarity to tissues, and their ability to form scaffolds for various tissues and their unique ability to tune the physical and mechanical properties according to the desired application.

The most commonly employed areas for hydrogels include bone tissue, cartilage tissue, vascular tissues, meniscus, tendon, skin, cornea, and soft tissues. Hydrogels have been used as scaffolds for fabrication or repair of the lost tissue (in cases where there is a loss of function) [103].

### 8.1. Bone and Cartilage Tissue Engineering 

Hydrogel matrices containing biological materials such as cells and growth factors with potential osteogenic and angiogenic potential and have been used for repair of large bone defects. Bone is a vascularized tissue and has the potential to heal naturally. Employment of 3D matrices will help in promoting cellular growth, which is an essential factor for bone regeneration. Three-dimensional cell culture hydrogel scaffolds have also been used for cartilage tissue engineering owing to their high-water content. Cartilage has limited ability for self-repairing, thereby prompting the researchers to look for an alternative substitute that can repair cartilage defects. The hydrogel class of materials has been looked upon as ideal substitutes that can repair cartilage defects due to their mechanical properties, swelling ability, and lubricating behaviour, which mimics the ECM of the articular cartilage. Hydrogels can encapsulate stem cells and can be loaded with growth factors, proteins that are essential for the promotion of cell differentiation. Hydrogel scaffolds have shown promising results in tissue engineering of bone and cartilage and seem to be an effective strategy for bone and cartilage tissue repair. According to Liu et al. (2017), among all of scaffolds for tissue-engineering applications, injectable hydrogels have demonstrated great potential for use as three-dimensional cell culture scaffolds in cartilage and bone tissue engineering, owing to their high water content, similarity to the natural extracellular matrix (ECM), porous framework for cell transplantation and proliferation, minimal invasive properties, and ability to match irregular defects [104].

### 8.2. Functional Bimolecular Delivery Systems

Articular cartilage is an avascular tissue that covers the weight-bearing areas of the joints. It is formed of chondrocytes, which are an organized population of cells found in cartilage connective tissue. They are the only cells located in healthy cartilage and are responsible for both synthesis and turnover of the abundant ECM. Due to the lack of access to blood supply, the articular cartilage has a limited ability to self-heal, and repair of the cartilage defects poses a major challenge.

Tissue engineering represents a promising mode of treatment of articular cartilage defects and osteoarthritis therapy. The collaborative and mutualistic activity of the mesenchymal stem cells (MSCs) and three-dimensional scaffolds play a crucial role in articular cartilage regeneration. However, this approach has limitations, such as poor mechanical properties of the engineered cartilage. Articular cartilage repair requires the delivery of bioactive growth factors and the collaboration of mesenchymal stem cells and scaffolds to contribute to collagen regeneration. Direct supply is limited by several factors, such as rapid degradation and need of dosages that are larger than that of an equivalent compound present in the body. There are also immune responses, inflammatory responses, and possibility of dissemination to non-target sites that may impair their therapeutic action. Synergic combination of biochemical factor-controlled delivery with other novel technologies—such as nanotechnology, mechanical stimulation, and other advanced techniques—offers a promising approach to controlled delivery of functional biomolecules.

To address this concern, tissue engineering involving modifiers that can influence the process, such as mechanical stimulation and functional biomolecule delivery systems (BDS), which represent a promising innovation to improve the regeneration process. The microenvironment of the tissue repair process involves a combination of many signals expressed in the biological system in response to injury. This concept should be considered during the construction of 3D scaffolds for regenerative studies. The need to use controlled delivery systems for cartilage repair (to control the release only where required) seems to provide a controlled release of bioactive molecules to specific target sites and overcomes the delivery problems [105].

Recently, many types of research focused their attention on the design of BDS, aiming to functionally improve scaffolds by combining MSCs with innovative 3D biomaterials and several biomolecules to be incorporated within the scaffolds. The latter, once released, should be able to guide the cells toward the chondrogenic stable phenotype, to achieve the proper mechanical properties.

Therefore, biomolecule delivery systems (BDS) can be considered a smart platform that can not only withstand external stresses but actively participate in the regeneration process through the release of biomolecules and bioactive factors. Functional biomaterials and BDS are characterized by the ability to control biological environment by providing appropriate three-dimensional settings and biochemical stimuli, which can significantly contribute to overcoming the limitations of conventional approaches [106].

Hydrogels seem to be suitable systems for molecular imprinting of biomolecules and for producing bioactive matrices. Hydrogels synthesized of natural polymers seem to have promising results. Three-dimensional printing technologies are a major area of development, considering their ability to deliver and deposit biomaterials in a much effective way, which is critical for tissue engineering.

### 8.3. Hydrogels for Three-Dimensional Cell Culture

Hydrogel systems, due to their hydrated polymer networks and similarity to native tissues, can be designed to control the cell fate and function, which is a fundamental concept in the field of tissue engineering. Hydrogel scaffolds have been used for 3D cell culture to act as a carrier of cellular contents for homogenous cell loading. Cells in the tissues are surrounded three-dimensionally by extracellular matrix in a complex microenvironment. Therefore, the biomaterials used as vehicles for carrying cells should replicate a similar environment found in natural tissues. Synthetic hydrogel systems have a reputation of being biocompatible, biodegradable, and can mimic the environment of natural tissues which serves as a platform for advanced applications such as tissue-engineered constructs. These constructs will provide a guide/template and a network into which cells exhibit adhesion, migration, and proliferation. The hydrogels also provide structural equilibrium allowing the cells to self-assemble. This system has a wide range of applications in cell biology, cancer biology, high-throughput drug screening, and tissue engineering. Hydrogel systems show promising results for three-dimensional cell culture and may find various applications in the field of regenerative medicine [107].

### 8.4. Hydrogels for Self-Healing

The specialized polymer hydrogels constructed from a network of several crosslinked polymer chains have been used for healing purposes in biomedical applications. The ability of these polymers to restore the morphological characteristics and mechanical properties of the damaged tissues resulting from injury and repeated damages makes them one of the most preferred choices of biomaterials used in wound healing. These classes of hydrogels possess the property to form new bonds when old bonds are broken within the materials by multi-mechanism interactions, and they can also sense environmental changes and adapt to them by fixing their properties and, therefore, the manner they operate.

Self-healing hydrogels are synthesized through dynamic covalent bonds and non-covalent interactions such as hydrogen bonds, electrostatic interaction, and hydrophobic interaction. According to a review by (Y. Liu & Hsu, 2018), self-healing hydrogels are classified as robust and soft hydrogels based on their mechanical properties in biomedical applications. Robust self-healing hydrogels are used as soft robots (such as implantable or wearable biosensors) with extended lifetime and mechanical performance due to repairing of damage or fatigue [108]. Soft self-healing hydrogels are used in cell/drug delivery and 3D printing due to injection through narrow needles and retention at target sites. Self-healing hydrogels encompass a wide range of medical applications like wound healing [109,110,111,112], surface coating, 3D printing [111,112], tissue engineering [113,114], and regeneration, drug delivery [115,116].

Smart hydrogels have also found application in the management of myocardial infarction. In a study conducted by (Gaffey et al., 2015), the self-healing hydrogel formed through host-guest interactions of adamantine- and β-cyclodextrin-modified hydroxyapatite (HA), was injected into the ischemic myocardium encapsulating endothelial progenitor cells (EPCs), a significant increase in vasculogenesis was noted with the hydrogel encapsulating EPCs [114].

### 8.5. Meniscus Tissue Engineering 

Meniscal tissue has a limited capacity to regenerate once they are damaged. The injectable hydrogel-based systems have provided an alternative to conventional meniscus treatment by being minimally invasive. The meniscus plays an essential role in maintaining the homeostasis of the knee joint, and tissue engineering approaches are of great importance to repair and regenerate damaged meniscus tissues in this area [117]. Enzyme-based methods were employed to fabricate tissue adhesive hydrogels for meniscus repair. Specific body tissues, such as cartilage and meniscus, have limited or no blood supply, and this makes them incapable of healing if they are damaged. By injecting a hydrogel loaded with repair cells or drugs into the damaged area, it may help in stimulating tissue regeneration.

### 8.6. Application of Injectable and Dynamic Hydrogels for Treatment of Intervertebral Disc Degeneration: Hydrogels as Efficient Nucleus Pulposus Replacement for Intervertebral Disc Repair, Substitution and Regeneration Possibilities

Chronic back pain is a disability, which is often linked to intervertebral disc degeneration. It is predominantly a middle-aged issue, and 50% of the population have degenerative changes in the cervical spine by middle age, and intervertebral disc degeneration is the most common cause of back pain. This disorder is also termed as degenerative disc disease (DDD). The first phase of the degenerative process usually involves the nucleus pulposus. Rapid recovery and regeneration of this structure will help to prevent further degradation of the annulus fibrosus. Advances in the development of regenerative medicine and tissue engineering have allowed the use of scaffolds aided with cells and growth factors and have opened up new possibilities for repair strategies. Injectable, bio-adhesive hydrogel has shown great potential to serve as a synthetic replacement for the nucleus pulposus of the intervertebral disc and has been used for regeneration of the nucleus [118].

The intervertebral disc, which is also called the intervertebral fibrocartilage, is located between the adjacent vertebrae in the vertebral column. The symphysis, which is a joint between the vertebral bodies functions as a ligament to keep the vertebrae together and provide a wide range of motion to the spine as a whole. This allows the cartilage to function as a shock absorber between each of the vertebrae in the spinal column by keeping the vertebrae separated when there is an impact from activity. The complex structure of the spine, which comprises both hard and soft tissues, imparts high load-bearing properties while still allowing motion and cushioning.

The primary components of the intervertebral discs are annulus fibrosus and a nucleus pulposus. It is a complex structure with periphery made of annulus fibrosus, which surrounds a jelly-like core (nucleus pulposus). The nucleus pulposus forms the core part and the locus of the vertebral disc. The annulus confines and contains the nucleus and forms the periphery. The function of the nucleus is to transfer the compressive loads to tensile loads in the annulus fibrosus as the annulus is not designed to take compressive loads primarily. The central part of the nucleus pulposus is composed of gelatin-like mass and a hydrated matter that consists of water, as well as a loose network of collagen fibers that resists compression.

The ability of the vertebral disc to withstand high forces of compression and torsion is due to the elasticity of the inner core structure. This hybrid structure also allows flexion, rotation, cushioning and protecting the function of the healthy spine. As ageing progresses, the body’s discs become stiffer due to dehydration of the structural components, causing the disc to be unable to adjust to compression. Therefore, there is a predilection for the nucleus pulposus to herniate and leak out of the disc space, causing inflammation of the nerve roots next to the disc, which leads to compression of the nerve through the protective layer of annulus fibrosus.

The development of naturally inspired solutions for the regeneration of the intervertebral disc plays a significant role in the development of treatment strategies for IVD degeneration totally in contrast to surgical problems for the management of IVD in the earlier past. This disease is highly prevalent among working class individuals and is usually triggered by acute physical trauma or prolonged intervertebral disc mistreatment, which aggravates the disease and provokes a cascade of degenerative changes that lead to chronic degenerative disorder.

When the intervertebral disc is intact, the loads are transferred from the NP to the annular walls in the periphery. When the nucleus is compromised, the loads are purely compressive on the walls resulting in annular buckling and degeneration. The current strategies for treatments of IVD degenerative disorders are ineffective in the longer-term and also may tend to spread to the adjacent intervertebral discs. Therefore, regenerative strategies are needed to compensate for the degeneration and allow for the healing and regeneration of damaged tissues, which can stop the progression of the disease.

The ideal candidate for nucleus replacement should have properties similar to the natural nucleus—highly hydrated, viscoelastic, inert, space-filling (even end-plate load distribution). Surgically, the material used for the regeneration of the nucleus should also be injectable, radiopaque and revisable, resist extrusion/expulsion, minimally invasive, and should not cause any damage to the annulus as much as possible.

Hydrogels are two-phased with water matrix supporting and surrounding a continuous polymer network: conformable, tough, viscoelastic, permeable, and mostly made up of water. Natural origin hydrogels have received a lot of interest in the literature, especially hydrogels that can be processed but not synthesized, thereby reducing the production costs significantly. They also have a low level of cytotoxicity, and a wide range of possible tissue engineering applications with suitable biological properties, such as bioactivity and biodegradability, while being available for cellular remodelling and cell adherence.

Poly (vinyl alcohol), which is prepared by hydrolysis of poly (vinyl acetate), is commonly used for commercial purposes such as textile industry, cosmetics industry, and adherent in paper industry. These classes of materials have seen increasing biomedical applications such as ophthalmic lubricant, artificial sponges, cartilage and meniscus replacement, nerve guides, etc. They are generally considered non-toxic, blood compatible, non-biodegradable, and hydrophilic.

### 8.7. Combination Therapy for Tissue Engineering Scaffolds: Cell-Seeded Scaffolds for Nucleus Pulposus (NP) Regeneration and Annulus Fibrosus (AF) Regeneration

The literature on this method of engineering for IVD regeneration suggests it is possible to regenerate the NP alone, but extensive efforts have been made in the recent past for simultaneous NP and AF. The metabolism of intervertebral discs is very slow, which leads to a longer regeneration time. Therefore, it is crucial that hydrogels have a degradation that is equal or greater so that they match the time frame. Natural origin hydrogels can serve as suitable materials that have been proposed in literature. Some of the commonly used hydrogels for regeneration of nucleus pulposus are based on the following raw materials: alginate [119,120], chitosan [121,122,123], collagen [124], gellan gum [125,126], and hyaluronic acid [127,128].

As per the literature, polysaccharides seem to be an ideal substituting material for the replacement of NP because they are cost-effective. However, due to the lack of vascular supply in the tissue, the degradable material, must allow cell adhesion and proliferation while not interfering with the natural angiogenesis. Gellan gums may be an alternative for the synthesis of hydrogels due to their non-angiogenic potential and may be able to provide a suitable environment for chondrocyte differentiation. Collagen could also serve as alternative to polysaccharides, even though it may be expensive and has shown excellent results in vitro regarding NP native-like ECM production, possibly due to its natural properties to mimic the ECM [129].

With the advances in the field of regenerative medicine and biomaterial engineering, in the future, there seems to be a possibility to produce 3-D printed custom-tailored AF scaffolds based on the host intervertebral geometry. The newer trends in manufacturing 3-D scaffolds will allow expanding the applicability of hydrogels into a broader range with specific details at a lower cost and a shorter time, which will benefit the treatment outcomes.

Synthetic hydrogels also serve as alternatives for fabrication. The physical and chemical properties can be tunable, and the degradation profile can be controlled according to the desired tissue regeneration rate. They can also easily blend with the polymers with enhanced mechanical properties that broaden even more than the normal, giving the hydrogel a higher viscoelastic nature. The synthetic hydrogels have a low risk of immunogenicity, infection, and toxicity concerns since they are made of known molecules which, when pure, exhibit no danger in all likelihood. Examples of synthetic hydrogels used for NP tissue engineering are polyethylene glycol, polyvinyl alcohol, and polyvinyl pyrrolidone. According to van Uden S. Silva-Correia, Oliveira J et al. (2017), the limitation of the synthetic hydrogel is that they lack bioactivity, which is uncharacteristic of the hydrogels from natural origin, and their manufacturing process is, in general, economically less attractive; these are two crucial factors for a tissue engineering application [129].

Even though natural-origin materials lack the diversity of properties, synthetic materials lack economically-viable options. The exciting possibility, however, is to use a natural-based material that can be altered and refitted with synthetic polymers to achieve the advantages of both types of materials. This concept needs future research on using combination therapy to improve the properties of the synthesized hydrogels.

Irrespective of all the current limitations, injectable hydrogels hold great promise for nucleus replacement strategies in intervertebral disc regeneration treatment. The edge and superiority of hydrogels due to properties—such as hydration, permeability, viscoelastic nature, minimal invasiveness, and being easily revised—make these classes of materials stronger candidates for regenerative medicine, and since it is critical for a surgical application that the material is injectable, hydrogels seem to be promising for future research. The future recommendations on the regeneration of the IVD should include patient-specific therapies and allow the development of scaffolds or constructs that fit the patient’s needs in a meticulous and pinpoint manner.

However, long term degradation profiles, quantitative determination of hydrogel ageing, and further in vivo safety and longevity studies will be required to evaluate the material when used for a longer-term.

### 8.8. Hydrogels for Drug Delivery

Hydrogel drug delivery systems can therapeutically have a beneficial outcome in clinical practice. They can provide spatial and temporal control over the release of various therapeutic agents [130]. Conventional drug usage often requires high dosages or repeated administrations to produce a therapeutic effect and has the disadvantages of side effects and toxicity concerns. Since the hydrogels can be tuned for its physical properties and mechanical properties and degradability, they serve as a platform in which various physicochemical interactions with the encapsulated drugs controlling their release. These drug delivery systems can control how the drugs are available to cells and tissues over time and in space which has been the most appealing part and has been used in many branches of medicine including cardiology, oncology, immunology, and pain management. Since they are formed in aqueous solutions, the risk of drug denaturation and aggregation upon exposure to solvents is minimized [130]. The cross-linked network of the polymeric structure of these hydrogel systems impedes penetration of various proteins and is believed to protect the bioactive therapeutics from premature degradation. Hydrogel-based drug delivery systems provide controlled drug release, and the impact of these systems is only going to rise in the future and is a promising platform for local drug delivery [115,116].

### 8.9. Skin Tissue Engineering

Hydrogels are considered suitable and ideal materials for epidermal tissue engineering and for the regeneration of skin, due to their unique combination of biological and physical properties, including biocompatibility and biomimetic nature, as well as on-site cross-linking capacities, and adjustable mechanical, swelling, and degradation properties [131]. Hydrogel scaffolds serve as matrices/scaffolds on which cells are entrapped which allow them to grow and proliferate and in turn repair the damaged tissue. Presence of biologically active materials encourages cell adhesion and migration. This application of hydrogels in skin tissue engineering has been mainly developed because of the drawbacks associated with the use of autografts and allografts where the donor site suffers from pain, recurrent infections, scarring over a while. Tissue-engineered skin replacements have found widespread applications in wound healing, especially in the case of burns, where the major limiting factor is the availability of autologous skin [131,132].

### 8.10. Tendon Tissue Engineering

Tissue engineering strategies have been used to improve tendon repair healing using the scaffolds, growth factors, cell seeding, or a combination of any of these approaches. Due to the acellularity of the tendon tissue, the regenerative capacity of tendon tissue is limited. As a result, current treatments for tendon injuries and tears often leave healed tendons with scar tissue that possesses compromised mechanical properties and has high risks of subsequent injuries and damages. Tissue engineering has emerged as a method to enhance tendon healing and regeneration using tissue engineering scaffolds, cells, and other biological factors. Scaffolds have been the most investigated strategy to date and injectable tendon hydrogels have been identified as potential scaffolds for guided tissue regeneration. The hydrogels are believed to potentially direct the collagen matrix of the tendon directly to the injury site, stimulating cell migration and healing. It can promote faster healing, allowing speedier recovery after common orthopedic injuries. The advantages of these hydrogel scaffolds are that they can be delivered non-invasively into a zone of damage, the scaffold polymerizes in vivo at body temperature. It acts as a carrier of cells or growth factors and conforms to injured tissue space/defect, provides a supportive nanostructure of collagen fibres that give stability to the structure, and aids in faster healing [133,134].

### 8.11. Cornea Tissue Engineering

Corneal disease is a serious condition that can cause clouding, distortion, scarring, and eventually blindness. Corneal disease is the second major cause of blindness [135]. Current treatment protocols revolve around corneal transplants, which are limited in supply. Hence, there is a need for alternative treatment methodologies to meet the rising demand for corneal replacements. The development of hydrogel-based polymeric bioscaffolds provides suitable alternative for corneal repair and regeneration. Most of the biomaterials which are currently available and are under investigation lack the biochemical composition of the native cornea. Xenografts are extracted from porcine and bovine corneas and have been used with considerable success post decellularization for corneal repair. However, they have several limitations, such as immune rejection and a major risk of disease transmission. Native cornea regeneration has received a great deal of attention, which uses tissue-engineered constructs by employing hydrogels and biopolymeric materials.

Tissue-engineered constructs used for corneal repair must mimic the native cornea to be able to perform normal physiological functions, which are critical for the maintenance of the living tissue. According to Kishore et al., 2016, preservation of the biochemical composition and tissue structure of the native cornea by employing optimal decellularization methods appears to be a promising approach for corneal tissue engineering [135]. Two types of hydrogels are used for this application: collagenous and non-collagenous hydrogels. Collagen matrices formed as gels, foams, and sponges are used for corneal applications. A tissue-engineered collagen sponge matrix has been shown to maintain the phenotype of human corneal cells [135]. Non-collagenous materials that include gelatin, keratin, chitosan, and silk have also been used.

Ahearne and Mark (2014), fabricated and tested a biomimetic hydrogel derived from the corneal extracellular matrix (ECM) to be used for corneal tissue engineering and demonstrated that hydrogels could be used for engineering corneal tissue. Hydrogels have the transparency to support the maintenance of functional keratocytes to preserve and de novo collagen tissue synthesis, and also to compensate for, and thereby prevent, the swelling rate of the native cornea to preserve and distribute the water content [136].

### 8.12. Hydrogel Prosthesis

Biomaterials such as hydrogels play a very decisive and distinctive role in the prosthetic rehabilitation of lost parts in the body. The hydrogel prosthesis is intended to interact with biological systems and is designed for implantation or incorporation with the living systems because the diffusion of oxygen through the hydrogel is comparatively higher than other material and hence can be stable near living tissues [137,138].

The biomaterials which are employed for the prosthetic purpose should be biocompatible, dimensionally stable, and—when implanted into the living system—should exhibit optimal thermal conductivity. These classes of materials show less mechanical stress to the living tissue compared to alternative materials due to their hydrophilic nature and can maintain their three-dimensional structure since they contain varying amounts of water depending on nature and density, and hence they serve as ideal prosthetic biomaterials. Their flexibility can also be altered and can be physically tuned to mimic the natural tissues, which make them excellent materials for future biomedical applications. These hydrogel polymer systems can be explicitly engineered with desirable properties such as biodegradation, adequate mechanical strength, and also act as biological response modifiers which can function by passively enhancing the immunologic response to the tumour cells or actively by altering the growth and differentiation of tumour cells in the living system. Therefore, the science of biological therapy is expanding, and the therapeutic value of these biomaterials will only expand in the future.

### 8.13. Smart Hydrogels for 3D Bioprinting

3D printing and smart hydrogels is a potent combination of bioprinting functional 3D tissues. Hydrogel in bioprinting acts as a matrix that supports and regulates the cells encapsulated inside the matrix. At the current stage, computational models have been set up to assess hydrogel contraction and deformation due to cellular events such as migration, proliferation and traction, cellular concentration, and distribution.

The smart hydrogel’s unique ability to react to a stimulus will also create an impact on the cells encapsulated inside the hydrogel. Time is an additional factor essential to understand the dynamic cell–material interaction since the properties of hydrogels change with time and are considered a critical factor in determining cell fate and tissue regeneration modelling at a quasi-static state. It demonstrates the need to consider dynamic changes in substrate viscoelasticity properties when determining tissue morphogenesis. In the case of the smart hydrogel, the changes in properties of smart hydrogel will affect the maturation of engineered tissue, and this remains an area for future investigation.

### 8.14. Smart Hydrogels as Actuators 

One of the unifying features amongst stimuli-responsive hydrogels is their ability to undergo reversible, discontinuous, and significant volume changes when subjected to external or internal stimuli. This ability of smart hydrogels to exhibit reversible ‘on-off’ swelling behaviour in the presence of physiologically relevant cues has been utilized to develop in vitro models mimicking various attributes of living systems, such as motility and actuating functions. Stimuli-responsive hydrogels have been used in designing micromanipulators, sensors, and optical devices with tunable focal length to control the flow of liquid in microfluidic devices. The concept of using stimuli-responsive hydrogels as actuators have gained vast attention. Their physical property/characteristic to undergo substantial volume changes when a stimulus is applied, and their capacity of being actuated, enrich the functionalities that can be developed by each actuator and may also facilitate the design of microfluidic devices. However, this technology is still at an early stage of development, and these smart hydrogels have immense potential to be applied in various microengineering products and biotechnological advancements [139,140].

## 9. Smart Hydrogels: Newer Advances 

### 9.1. Biomolecular Responsive Hydrogels as Smart Sensors

In recent years, numerous studies have emphasized on the use of smart biomimetic hydrogels in building biochemical sensors. The inherent ability of these water-based corporeality to respond to its surrounding stimulus by undergoing phase change mechanisms and transmitting biological data to these sensors has led to a tremendous improvement in the treatment of many diseases. One such study includes the use of enzyme-free hydrogels specially made to respond to metabolic changes by acting as chemo-mechanical transducers in piezoresistive sensors. The principle chiefly focuses on evaluating the hydrogel’s response to varying amounts of glucose in the solution, which in turn alerts the sensors to help identify metabolic changes [140]. With such biosensors, the perpetual need for measuring constant blood sugar levels can be achieved in diabetic patients.

In recent studies, scientists have described methods for the fabrication of economical, yet promptly responsive, hydrogels by creating microscopic, smart hydrogel pillar-like edifices with enhanced surface area to volume ratios built within microfluidic channels. When these pillars encounter the target, molecules present in the solutions, they shrink and swell, leading to an alteration in resistance, which can be analyzed by the use of a potentiostat [141]. However, the inconsistent swelling properties of smart hydrogels remains as a challenge for their use in biosensing applications.

### 9.2. Hydrogels in Cardiac Tissue Engineering

The number of people affected by cardiac problems every year is rising globally, and one of the many actions taken to address this broad concern is employing tissue engineering techniques for cardiac tissue repair and regeneration. Although there are certain restraints in the ability of human cardiomyocytes to regenerate completely, several tissue engineering strategies using hydrogels have been deployed over the years to overcome this limitation.

One such step is the development of a blend of collagen-fibrin based hydrogel, seeded with cardiomyocytes derived from human pluripotent stem cells, which revealed that hydrogel protein content and the population of cell seeding plays a critical role in myocardial tissue regeneration [142]. Medical literature shows the development of 3D bio-printed gelatin-based hydrogels, which are micro-channeled to help promote heart cell growth by maintaining native cardiomyocytes while at the same time utilizing mesenchymal stem cells to affect cardiac regeneration [143]. Nonetheless, more extensive research must be conducted to overcome contemporary drawbacks in cardiac tissue growth.

### 9.3. Hydrogels in Neural Tissue Engineering

Treatment of damaged or injured neural structures in the central nervous system (CNS) remains a difficult and challenging task for physicians and surgeons, universally attributed to the highly complex structure and reduced regeneration potential of nerve cells. Numerous natural and synthetic hydrogel-based biomaterials are studied currently in vivo to promote neural tissue growth by acting as nerve guides, conduits, or tubulation [144]. Among them, the use of porous hydrogel matrices for nerve repair is widely used in the past due to their increased stability, allowing them to sustain tissue ingrowth and organization for longer spans. Although still in its outset, neural tissue engineering by means of hydrogel matrices holds great outlook by focusing on two related issues: (1) the design of polymer matrices with the ability to control biological actions and improvement in tissue-building capabilities for effective local tissue repair, and (2) the initiation of cell-based technologies for the fabrication of hybrid biomaterials or cell-engineered biological constructs [145].

### 9.4. Hydrogels as Immuno-Isolation Devices

The notion of immuno-isolation was developed to protect the foreign graft material form the host immune response and to avoid complications occurring due to immune suppression. Immuno- isolation devices were designed to establish a physical barrier with the newly grafted tissues to limit their contact with host immune cells. For a graft material to be viable and functional, the device should be fabricated to allow sufficient diffusion of nutrients, endocrine factors, and oxygen to the implanted tissues to sustain extensive interaction with the environment [146]. Researchers have utilized the excellent biocompatibility and structural properties of smart hydrogels to mimic the extracellular matrix. To create injectable hydrogel matrices for delivering islet cells in the site of transplantation in the treatment of type 1 diabetes [147].

Encapsulating of the islet cells in hydrogel microspheres (microgels) before transplantation into diabetic recipients will establish an adequate immune-isolation barrier to depreciate allogeneic rejection. Synthetic hydrogel macromers like PEG-4MAL (4-arm polyethylene glycol terminated with maleimides) can serve as an ideal candidate for immuno-isolation applications. They can be easily altered with thiolated bioactive molecules, allowing precise control of the islet microenvironment [148]. Although the advancements in cell therapy, nanotechnology, biotechnology, genetic engineering, and immunology have recognized potential means of using hydrogels in accomplishing long-term viability and functionality of transplanted islet cells, many hurdles are yet to be crossed, such as islet cell apoptosis, the need of immunosuppressive drugs, and managing immune reactions for successful therapeutic results [147].

### 9.5. Magnetic Induced Hydrogels 

Magnetic nanoparticles are used in various diagnostic applications both in vitro and in vivo due to their low interference with biological processes. These nanoparticles tend to lose their magnetism due to their small size when the magnetic field is eliminated. Current use in biomedicine includes cell therapy, tissue repair, drug delivery, and magnetic resonance imaging (MRI), and other applications [149]. One of the main applications of magnetic hydrogels is for targeted chemotherapeutic drug delivery to tumours. It allows tissue-specific sites to be targeted, which in turn reduces the potentially toxic side effects on surrounding unaffected tissues, thus increasing the efficacy of the drug. Another promising application involves specifically targeting diseased tissues using hydrogels that can later be imaged with an MRI, enhancing the image contrast and improving the overall efficiency of patient diagnosis [150].

Hydrogel research consolidates magnetic nanoparticles within their three-dimensional network. These are known as ferrogels because of the embedded iron oxide particles in the matrix [151]. The movement of the particles is regulated by the magnetic field besieging them, which leads to gel deformation and thus controls the shape and elasticity of hydrogels. These movements are mapped using computer simulations, and the magnetic responses and physical characteristics of ferrogels are analyzed. Weeber and Holm suggested the use of software known as ESPResSO (extensible simulation package for research on soft matter systems), which uses the computer to track the position and orientation of nanoparticles suggesting that several factors like crosslinking, resiliency, and elasticity affect the deformation of ferrogels [152].

## 10. Current Status of Hydrogels Concerning their Synthesis and Fabrication

Smart hydrogels are a remarkable class of materials that could be programmed to react to specific stimuli such as pH, temperature, light, electric, and magnetic field. Hydrogels are thought of as the most suitable materials for matching the chemical, physical, and mechanical properties with the natural extracellular matrix (ECM). Hydrogels are commonly developed as a candidate for implantable artificial muscles, cell scaffolds, and tissue organs [153]. Numerous strategies applicable to the implantable biomaterials highly depend on the fabrication of functional hydrogels. Novel approaches in hydrogel design have revitalized this field of biomaterials research. However, the direction of where smart hydrogels will go in the future will need to be addressed. All the scientific evidence seems to indicate that basic and translational research in hydrogels has a bright future. Numerous new designs—e.g., involving protein domains containing non-canonical amino acids—attempts to control the morphology of self-assembling peptide fibres, artificial glycoproteins for controlling cell responses, hydrogels as the building material for micro chemotaxis devices, enhanced use of DNA recognition motifs, and improved synthetic methods demonstrate the versatility of the manufacturing approaches of smart hydrogels [154].

Hydrogels, in most cases, are homogeneous materials with an amorphous structure. Natural bio-tissues often possess well-defined hierarchical and anisotropic structures at variable scales, such as muscles, skin, and articular cartilage. These structural features of the bio-tissues are favourable to highly elaborate functions of living organisms [155]. Thus, control of hydrogel structure exhibiting complex functionalization at different scales is necessary for biologically-related applications. However, until now, the precise design of the hydrogels remains a significant challenge for scientists. Many techniques have been developed to engineer three-dimensional hydrogels with unique geometry and specific patterns depending on desired applications. The production of hydrogel structures with three-dimensional ordered patterns, array, or complex geometry with broad scales ranging from sub-micrometres and micrometres to millimetres is especially challenging [156]. Both the size and morphology features of the hydrogels should highly be matched with when considering various applications. Each method possesses its advantages suitable for a particular use. However, it should be noted here that, in many cases, the reported hydrogels microstructures are only focused on dimensions and morphologies, without considering the strength and stability. Until now, the strength of the synthesized hydrogels is still weak in comparison with natural biological tissue [157].

Hydrogels will have a vital role to play in the field of biomedicine and nanotechnology. The future success of hydrogels is based on the synthesis of new polymers or on the modification of natural polymers to solve specific biological and medical difficulties [157]. Most of the scientific literature shows that the studies on hydrogels have a bright future. Investigation of these biomaterials will provide new approaches to hydrogel designs. New ideas on the design of hydrogels with substantially enhanced mechanical properties, super porous and comb-type grafted hydrogels with fast response times, self-assembling hydrogels from hybrid graft copolymers with property-controlling protein domains, and genetically engineered triblock copolymers are just a few examples of hydrogel biomaterials with a bright future [158].

New techniques with in situ cell encapsulation ability for synthesizing smart hydrogels with high strength should be developed with consideration of these drawbacks. Recently, 3D bioprinting has opened up a novel way for engineering high strength hydrogels with complex shapes, dimensions achieved to sub-micrometres or nanometers, which is even more desirable. Furthermore, clinical research should be more focused on producing nanoscale hydrogels constructs. There will be setbacks on the way forward, but the scientific and translational potential of hydrogel biomaterials makes it a confident material in predicting a bright future.

## 11. Limitations of Hydrogel Systems 

The hydrogel systems have played a significant role in developing tissue engineering scaffolds for the past decade. Hydrogels represent to be an interesting material for regenerative medicine and tissue engineering applications. As responsive ‘smart materials,’ hydrogels mimic three-dimensional tissue environments. It allows the timely release of growth factors, proteins, and peptides, other nutrients to ensure proper tissue growth. However, there are certain limitations to using hydrogel systems. Hydrogels show difficulty in handling and loading since they tend to be fragile. Hydrogels tend to exhibit an inhomogeneous crosslink density distribution, which is known as spatial inhomogeneity, and this property is not desirable because it reduces the strength of the hydrogels. Hydrogels are non-adherent, so they may need to be secured by a secondary dressing, and they are also not very absorptive, so they are not preferred for treatment of moderate to high exudation wounds. Hydrogels used as contact lenses cause lens deposition, hypoxia, dehydration, and eye reaction due to immune responses and inflammatory reactions. These classes of materials also have a low mechanical strength due to soft structures and tend to be delicate. Hydrogels are biocompatible and biodegradable. The issue related to the degradation of hydrogels has received much attention in the recent past even though hydrogels usually undergo degradation hydrolytically and enzymatically withy time. Hydrogels made of natural polymers usually are biodegradable and most often biocompatible. However, one limitation of the hydrogels made of natural polymers is that they are difficult to isolate from the biological tissue since they mimic and resemble the natural tissue they replace when used in tissue engineering, and they will also have restricted versatility.

On the other hand, synthetic hydrogels also have a limitation in that they may not be biocompatible or biodegradable. The other limitation that these systems possess is difficulty in sterilization. Sterilization of hydrogels can be more complicated than in other types of polymers due to the presence of water in these materials that can potentiate the harmful effects of the sterilizing processes. Despite the useful properties and phenomenal potential of hydrogels for regenerative medicine, several challenges persist, especially regarding their sensitivity to the conventional sterilization protocols. Any biomaterial must withstand efficient sterilization to obtain approval from regulatory organizations and to proceed to clinical trials safely. Assurance of sterility reduces the incidence of medical device-related infections. It is essential to understand the impossibility and unattainable nature in predicting the outcome of various methods of sterilization to determine certain standard rules and protocols for sterilization considering the complexity of factors involved such as properties of materials, drug stability, and sterilization conditions. Hydrogels also have a drawback in that it is challenging to coordinate the degradation rate. The degradation profile of the hydrogels depends on the type of application. In situations such as for cell cultivation, biodegradable hydrogels usually act as a temporary extracellular matrix (ECM) until replaced by a new tissue and the faster degradation profile would be suitable. For drug delivery applications, we need the hydrogels to remain in the target site for a much longer time to be able to deliver nutrients and bioactive molecules for new tissue growth. Biodegradable hydrogels will be degraded entirely after a certain period based on the designed polymer structure. Their persistent time ranges from days to several months. The advantage of biodegradable hydrogels in biomedical applications is that we do not have to remove the materials due to their complete degradation at the end. However, further research will be required to understand the nature of degradation and its relation to the applications in tissue engineering. Hydrogels also have a limitation that the mechanism of crosslinking affects release profiles, and there is a risk of toxicity posed by the chemical cross-linkers. However, Hydrogel systems continue to be one of the dynamic systems with properties that can be tuned and are useful in the fundamental understanding of the cell-matrix interactions, which are essential for promoting tissue regeneration.

## 12. Future Perspectives 

The past few decades have shown a paradigm shift in designs, fabrication techniques, properties, and applications of smart hydrogels in biomedical engineering. This change can be attributed to extensive research conducted in developing more sophisticated hydrogel-based matricing systems in the field of regenerative medicine. Current research is focused principally on altering the chemistry of biopolymers, leading to a significant improvement in the mechanical and biological properties of hydrogels. The features of hydrogels make them one of the pioneer components in healthcare applications. Repeated efforts were made to tailor smart hydrogels for specific applications by carefully investigating their surrounding microenvironments and utilizing these functional improvements for further exploitation.

Finally, despite significant advancements in bioengineering techniques for hydrogel fabrication, several parameters—like the degradation rates, reaction time, surface hybridization, microstructures, inflammation, and immunological response—of these materials demand a careful assessment to synthesize more cytocompatible hydrogels for tissue engineering applications. In the coming years, further improvement in their function and structure can lead us to a better understanding of cell-material interactions, allowing us to use a modular approach to generate new tissues. These hydrogels can alternatively provide a much safer and thriving modality in the treatment of numerous diseases.

## 13. Final Remarks and Conclusion 

This brief review on smart hydrogels attempts to focus on the timeline of developments that took place since their initial application in biomedical science. It outlines the essence of research conducted to modify their properties and create and establish new specific applications and the recent advances in regenerative medicine. The research in the next decade of regenerative medicine can continue to offer unprecedented advances within the field of tissue engineering. However, research results are yet to transfer their usefulness and clinical pertinence. This subject of ‘smart hydrogels for smart applications will require not only outstanding research but also teamwork between clinicians and researchers. Given the advances in research and looking into the future, new characterization and modelling methods will be needed to aid the systematic investigation of applying smart hydrogels in bioprinting. With interdisciplinary approaches, smart hydrogels will be intelligent biomaterials that can revolutionize the field of regenerative medicine in the future.

## Figures and Tables

**Table 1:** Overview of hydrogels and their applications

Hydrogel	Application	Reference
Temperature responsive hydrogels	Skin tissue engineering, wound covering, cell carriers	[131,132]
Light responsive hydrogels	Drug delivery, microfluidic devices	[115,116,130]
Electro responsive hydrogels	Membrane and implant-based drug delivery	[137,138]
Magnetic responsive hydrogels	Drug delivery, tissue repair, targeted MRI for disease diagnosis	[149,150,151,152]
pH-responsive hydrogels	Drug and protein delivery, 3D cell culture	[107,115,116,130]
Glucose responsive hydrogels	Immunoisolation devices	[146,147,148]
Biochemical responsive hydrogels	Smart sensors and actuators	[139,140,141]
Collagen-based hydrogels	Corneal, tendon tissue engineering	[133,134,135,136]
Injectable hydrogels	Bone, cartilage and meniscus tissue engineering, drug delivery, Osteo-arthritis therapy	[104,118,119,120,121,122,123,124,125,126,127,128,129]

## References

[1] Bacelar A.H., Cengiz I.F., Silva-Correia J., Sousa R.A., Oliveira J.M., Reis R.L. (2017). “Smart” hydrogels in tissue engineering and regenerative medicine applications. Handbook of Intelligent Scaffolds for Tissue Engineering and Regenerative Medicine.

[2] Panyam J., Labhasetwar V. (2012). Biodegradable nanoparticles for drug and gene delivery to cells and tissue. Adv. Drug Deliv. Rev..

[3] Hamidi M., Azadi A., Rafiei P. (2008). Hydrogel nanoparticles in drug delivery. Adv. Drug Deliv. Rev..

[4] Uhrich K.E., Cannizzaro S.M., Langer R.S., Shakesheff K.M. (1999). Polymeric Systems for Controlled Drug Release. Chem. Rev..

[5] Langer R. (1990). New methods of drug delivery. Science.

[6] Slaughter B.V., Khurshid S.S., Fisher O.Z. (2009). Hydrogels in regenerative medicine. Adv. Mater..

[7] Bettinger C., Borenstein J., Langer R. (2014). Microfabrication techniques in scaffold development. Nanotechnology and Regenerative Engineering.

[8] Lee K.Y., Mooney D. (2001). Hydrogels for tissue engineering. Am. Chem. Soc. Chem. Rev..

[9] Drury J.L., Mooney D.J. (2003). Hydrogels for tissue engineering: Scaffold design variables and applications. Biomaterials.

[10] Khademhosseini A., Langer R. (2016). A decade of progress in tissue engineering. Nat. Protoc..

[11] Leijten J., Seo J., Yue K., Trujillo-de Santiago G., Tamayol A., Ruiz-Esparza G.U., Khademhosseini A., Shin S.R., Sharifi R., Noshadi I. (2017). Spatially and temporally controlled hydrogels for tissue engineering. Mater. Sci. Eng. R Rep..

[12] Ratner B.D., Hoffman A.S. (1976). Synthetic Hydrogels for Biomedical Applications. Hydrogels Med. Relat. Appl..

[13] Burczak K., Gamian E., Kochman A. (1996). Long-term in vivo performance and biocompatibility of poly(vinyl alcohol) hydrogel microcapsules for hybrid-type artificial pancreas. Biomaterials.

[14] Jalili N.A., Muscarello M., Gaharwar A.K. (2016). Nanoengineered thermoresponsive magnetic hydrogels for biomedical applications. Bioeng. Transl. Med..

[15] Dong R., Pang Y., Su Y., Zhu X. (2015). Supramolecular hydrogels: Synthesis, properties and their biomedical applications. Biomater. Sci..

[16] Chung B.G., Lee K.H., Khademhosseini A., Lee S.H. (2012). Microfluidic fabrication of micro engineered hydrogels and their application in tissue engineering. Lab Chip. R. Soc. Chem..

[17] Koutsopoulos S. (2016). Self-assembling peptide nanofiber hydrogels in tissue engineering and regenerative medicine: Progress, design guidelines, and applications. J. Biomed. Mater. Res. Part A.

[18] Khandan A., Jazayeri H., Fahmy M.D., Razavi M. (2017). Hydrogels: Types, structure, properties, and applications. Frontiers in Biomaterials.

[19] Silva S.S., Fernandes E.M., Pina S., Silva-Correia J., Vieira S., Oliveira J.M., Reis R.L. (2017). Natural-origin materials for tissue engineering and regenerative medicine. Compr. Biomater. II.

[20] Wichterle O., Lím D. (1960). Hydrophilic gels for biological use. Nature.

[21] Zhu J., Marchant R.E. (2011). Design properties of hydrogel tissue-engineering scaffolds. Expert Rev. Med. Devices.

[22] Hoffman A.S. (2002). Hydrogel biomedical articles. Adv. Drug Deliv. Rev..

[23] Shi D., Shi D., Jiang G. (2010). An Introduction to Biomaterials.

[24] Khan F., Tare R.S., Kanczler J.M., Oreffo R.O., Bradley M. (2010). Strategies for cell manipulation and skeletal tissue engineering using high-throughput polymer blend formulation and microarray techniques. Biomaterials.

[25] Khan F., Tare R.S., Kanczler J.M., Oreffo R.O., Bradley M. (2013). Discovery and evaluation of a functional ternary polymer blend for bone repair: Translation from a microarray to a clinical model. Adv. Funct. Mater..

[26] Liu G., Ding Z., Yuan Q., Xie H., Gu Z. (2018). Multi-layered hydrogels for biomedical applications. Front. Chem..

[27] Ahmed E.M. (2015). Hydrogel: Preparation, characterization, and applications: A review. J. Adv. Res..

[28] Maitra J., Shukla V.K. (2014). Cross-linking in Hydrogels—A Review. Am. J. Polym. Sci..

[29] Fan C., Wang D.-A. (2017). Macroporous hydrogel scaffolds for three-dimensional cell culture and tissue engineering. Tissue Eng. Part B Rev..

[30] Eltom A., Zhong G., Muhammad A. (2019). Scaffold Techniques and Designs in Tissue Engineering Functions and Purposes: A Review. Adv. Mater. Sci. Eng..

[31] Jordan A.M., Kim S.E., Van De Voorde K., Pokorski J.K., Korley L.T.J. (2017). In situ fabrication of fiber reinforced three-dimensional hydrogel tissue engineering scaffolds. ACS Biomater. Sci. Eng..

[32] Wang J., Zhang F., Tsang W.P., Wan C., Wu C. (2017). Fabrication of injectable high strength hydrogel based on 4-arm star PEG for cartilage tissue engineering. Biomaterials.

[33] Lu T., Li Y., Chen T. (2013). Techniques for fabrication and construction of three-dimensional scaffolds for tissue engineering. Int. J. Nanomed..

[34] Satapathy M.K., Chiang W.-H., Chuang E.-Y., Chen C.-H., Liao J.-L., Huang H.-N. (2017). Microplasma-assisted hydrogel fabrication: A novel method for gelatin-graphene oxide nanocomposite hydrogel synthesis for biomedical application. PeerJ.

[35] Klouda L. (2015). Thermoresponsive hydrogels in biomedical applications: A seven-year update. Eur. J. Pharm. Biopharm..

[36] Klouda L., Mikos A.G. (2008). Thermoresponsive hydrogels in biomedical applications. Eur. J. Pharm. Biopharm..

[37] Koetting M.C., Peters J.T., Steichen S.D., Peppas N.A. (2015). Stimulus-responsive hydrogels: Theory, modern advances, and applications. Mater. Sci. Eng. R Rep..

[38] Qiu Y., Park K. (2001). Environment-sensitive hydrogels for drug delivery. Adv. Drug Deliv. Rev..

[39] Ghadban A., Ahmed A.S., Ping Y., Ramos R., Arfin N., Cantaert B., Miserez A., Ramanujan R.V. (2016). Bioinspired pH and magnetic responsive catechol-functionalized chitosan hydrogels with tunable elastic properties. Chem. Commun..

[40] Satarkar N.S., Hilt J.Z. (2008). Magnetic hydrogel nanocomposites for remote-controlled pulsatile drug release. J. Control. Release.

[41] Chen S.C., Wu Y.C., Mi F.L., Lin Y.H., Yu L.C., Sung H.W. (2004). A novel pH-sensitive hydrogel composed of N, O-carboxymethyl chitosan and alginate cross-linked by genipin for protein drug delivery. J. Control. Release.

[42] Topuz F., Buenger D., Tanaka D., Groll J. (2011). Hydrogels in biosensing applications. Compr. Biomater..

[43] Gu Z., Dang T.T., Ma M., Tang B.C., Cheng H., Jiang S., Dong Y., Zhang Y., Anderson D.G. (2013). Glucose-responsive microgels integrated with enzyme nanocapsules for closed-loop insulin delivery. ACS Nano.

[44] Wilson A.M., Justin G., Guiseppi-Elie A. (2010). Electroconductive Hydrogels. Biomedical Applications of Hydrogels Handbook.

[45] Gulrez S.K., Al-Assaf S., Phillips G.O. (2011). Hydrogels: Methods of Preparation, Characterisation and Applications. Progress in Molecular and Environmental Bioengineering—From Analysis and Modeling to Technology Applications.

[46] He L., Fullenkamp D.E., Rivera J.G., Messersmith P.B. (2011). pH-responsive self-healing hydrogels formed by boronate-catechol complexation. Chem. Commun..

[47] Sood N., Bhardwaj A., Mehta S., Mehta A. (2016). Stimuli-responsive hydrogels in drug delivery and tissue engineering. Drug Deliv..

[48] Buenger D., Topuz F., Groll J. (2012). Progress in Polymer Science Hydrogels in sensing applications. Prog. Polym. Sci..

[49] Li L., Scheiger J.M., Levkin P.A. (2019). Design and applications of photoresponsive hydrogels. Adv. Mater..

[50] Mano J.F. (2008). Stimuli-responsive polymeric systems for biomedical applications. Adv. Eng. Mater..

[51] Shantha K.L., Harding D.R.K. (2002). Synthesis and evaluation of sucrose-containing polymeric hydrogels for oral drug delivery. J. Appl. Polym. Sci..

[52] Lopes J., Fonseca R., Viana T., Fernandes C., Morouço P., Moura C., Biscaia S. (2019). Characterization of Biocompatible Poly(Ethylene Glycol)-Dimethacrylate Hydrogels for Tissue Engineering. Appl. Mech. Mater..

[53] Saini K. (2017). Preparation method, Properties and Crosslinking of hydrogel: A review. Pharma Tutor.

[54] Kim S.H., Chu C.C. (2009). Visible light-induced dextran-methacrylate hydrogel formation using (-)-riboflavin vitamin B2 as a photoinitiator and L-arginine as a co-initiator. Fibres Polym..

[55] Gyles D.A., Castro L.D., Silva J.O.C., Ribeiro-Costa R.M. (2017). A review of the designs and prominent biomedical advances of natural and synthetic hydrogel formulations. Eur. Polym. J..

[56] El-Sherbiny I.M., Yacoub M.H. (2013). Hydrogel scaffolds for tissue engineering: Progress and challenges. Glob. Cardiol. Sci. Pract..

[57] Bencherif S.A., Braschler T.M., Renaud P. (2013). Advances in the design of macroporous polymer scaffolds for potential applications in dentistry. J. Periodontal Implant Sci..

[58] Autissier A., Le Visage C., Pouzet C., Chaubet F., Letourneur D. (2010). Fabrication of porous polysaccharide-based scaffolds using a combined freeze-drying/cross-linking process. Acta Biomater..

[59] Ma P.X. (2004). Scaffolds for tissue fabrication. Mater. Today.

[60] Dehghani F., Annabi N. (2011). Engineering porous scaffolds using gas-based techniques. Curr. Opin. Biotechnol..

[61] Nam Y.S., Yoon J.J., Park T.G. (2000). A novel fabrication method of macroporous biodegradable polymer scaffolds using gas foaming salt as a porogen additive. J. Biomed. Mater. Res..

[62] Yoon J.J., Park T.G. (2001). Degradation behaviours of biodegradable macroporous scaffolds prepared by gas foaming of effervescent salts. J. Biomed. Mater. Res..

[63] Hutmacher D.W., Woodfield T.B.F., Dalton P.D. (2014). Scaffold design and fabrication. Tissue Engineering: Second Edition.

[64] Sequeira S.J., Soscia D.A., Oztan B., Mosier A.P., Jean-Gilles R., Gadre A., Larsen M., Cady N.C., Yener B., Castracane J. (2012). The regulation of focal adhesion complex formation and salivary gland epithelial cell organization by nanofibrous PLGA scaffolds. Biomaterials.

[65] Parrag I.C., Zandstra P.W., Woodhouse K.A. (2012). Fibre alignment and coculture with fibroblasts improve the differentiated phenotype of murine embryonic stem cell-derived cardiomyocytes for cardiac tissue engineering. Biotechnol. Bioeng..

[66] Huang G.Y., Zhou L.H., Zhang Q.C., Chen Y.M., Sun W., Xu F., Lu T.J. (2011). Microfluidic hydrogels for tissue engineering. Biofabrication.

[67] Khan F., Tanaka M., Ahmad S.R. (2015). Fabrication of polymeric biomaterials: A strategy for tissue engineering and medical devices. J. Mater. Chem. B R. Soc. Chem..

[68] Bryant S.J., Nuttelman C.R., Anseth K.S. (2000). Cytocompatibility of UV and visible light photoinitiating systems on cultured NIH/3T3 fibroblasts in vitro. J. Biomater. Sci. Polym. Ed..

[69] Hahn M.S., Miller J.S., West J.L. (2006). Three-dimensional biochemical and biomechanical patterning of hydrogels for guiding cell behaviour. Adv. Mater..

[70] Garg T., Singh O., Arora S., Murthy R.S.R. (2012). Scaffold: A novel carrier for cell and drug delivery. Crit. Rev. Ther. Drug Carr. Syst..

[71] Peppas N.A., Huang Y., Torres-Lugo M., Ward J.H., Zhang J. (2000). Physicochemical foundations and structural design of hydrogels in medicine and biology. Annu. Rev. Biomed. Eng..

[72] Kennedy J.F., Taylor D.W. (2003). Thermoreversible gelation of polymers and biopolymers. Carbohydr. Polym..

[73] Semenov A.N., Rubinstein M. (1998). Thermoreversible gelation in solutions of associative polymers. 1. Statics. Macromolecules.

[74] Okumura Y., Ito K. (2001). The polyrotaxane gel: A topological gel by figure-of-eight cross-links. Adv. Mater..

[75] Ito K. (2007). Novel cross-linking concept of polymer network: synthesis, structure, and properties of slide-ring gels with freely movable junctions. Polym. J..

[76] Haraguchi K., Takehisa T. (2002). Nanocomposite hydrogels: A unique organic-inorganic network structure with extraordinary mechanical, optical, and swelling/De-swelling properties. Adv. Mater..

[77] Gong J.P., Katsuyama Y., Kurokawa T., Osada Y. (2003). Double-network hydrogels with extremely high mechanical strength. Adv. Mater..

[78] Sakai T., Matsunaga T., Yamamoto Y., Ito C., Yoshida R., Suzuki S., Chung U.I., Sasaki N. (2008). Design and fabrication of a high-strength hydrogel with an ideally homogeneous network structure from tetrahedron-like macromonomers. Macromolecules.

[79] Shibayama M. (2012). Structure-mechanical property relationship of tough hydrogels. Soft Matter. R. Soc. Chem..

[80] Anseth K.S., Bowman C.N., Brannon-Peppas L. (1996). Mechanical properties of hydrogels and their experimental determination. Biomaterials.

[81] Brandl F., Sommer F., Goepferich A. (2007). Rational design of hydrogels for tissue engineering: Impact of physical factors on cell behaviour. Biomaterials.

[82] Wang W.Y., Pearson A.T., Kutys M.L., Choi C.K., Wozniak M.A., Baker B.M., Chen C.S. (2018). Extracellular matrix alignment dictates the organization of focal adhesions and directs uniaxial cell migration. APL Bioeng..

[83] Raab M., Discher D.E. (2017). Matrix rigidity regulates microtubule network polarization in migration. Cytoskeleton.

[84] Xiao L., Zhu J., Londono J.D., Pochan D.J., Jia X. (2012). Mechano-responsive hydrogels crosslinked by block copolymer micelles. Soft Matter.

[85] Sathaye S., Mbi A., Sonmez C., Chen Y., Blair D.L., Schneider J.P., Pochan D.J. (2015). Rheology of peptide- and protein-based physical hydrogels: Are everyday measurements just scratching the surface?. Wiley Interdiscip. Rev. Nanomed. Nanobiotechnol..

[86] Wang S.Q., Ravindranath S., Boukany P.E. (2011). Homogeneous shear, wall slip, and shear banding of entangled polymeric liquids in simple shear rheometry: A roadmap of nonlinear rheology. Macromolecules.

[87] Fanesi G., Abrami M., Zecchin F., Giassi I., Dal Ferro E., Boisen A., Marizza P., Grassi G., Bertoncin P., Grassi M. (2018). Combined Used of Rheology and LF-NMR for the Characterization of PVP-Alginates Gels Containing Liposomes. Pharm. Res..

[88] Van Tomme S.R., Storm G., Hennink W.E. (2008). In situ gelling hydrogels for pharmaceutical and biomedical applications. Int. J. Pharm..

[89] Ahmed T.A., Alharby Y.A., El-Helw A.R.M., Hosny K.M., El-Say K.M. (2016). Depot injectable atorvastatin biodegradable in situ gel: Development, optimization, in vitro, and in vivo evaluation. Drug Des. Dev. Ther..

[90] Rizwan M., Yahya R., Hassan A., Yar M., Azzahari A.D., Selvanathan V., Abouloula C.N., Sonsudin F. (2017). pH-sensitive hydrogels in drug delivery: Brief history, properties, swelling, and release mechanism, material selection and applications. Polymers.

[91] Shazeeb M.S., Corazzini R., Konowicz P.A., Fogle R., Bangari D.S., Johnson J., Dhal P.K., Ying X. (2018). Assessment of in vivo degradation profiles of hyaluronic acid hydrogels using temporal evolution of chemical exchange saturation transfer (CEST) MRI. Biomaterials.

[92] Li X., Kondo S., Chung U.I., Sakai T. (2014). Degradation behaviour of polymer gels caused by nonspecific cleavages of network strands. Chem. Mater..

[93] Li X., Tsutsui Y., Matsunaga T., Shibayama M., Chung U.I., Sakai T. (2011). Precise control and prediction of hydrogel degradation behaviour. Macromolecules.

[94] Pradhan S., Keller K.A., Sperduto J.L., Slater J.H. (2017). Fundamentals of Laser-Based Hydrogel Degradation and Applications in Cell and Tissue Engineering. Adv. Healthc. Mater..

[95] Unadkat H.V., Hulsman M., Cornelissen K., Papenburg B.J., Truckenmüller R.K., Carpenter A.E., Stamatialis D. (2011). An algorithm-based topographical biomaterials library to instruct cell fate. Proc. Natl. Acad. Sci. USA.

[96] Chen Q., Chen H., Zhu L., Zheng J. (2015). Fundamentals of double network hydrogels. J. Mater. Chem. B R. Soc. Chem..

[97] Xing L., Hu C., Zhang Y., Wang X., Shi L., Ran R. (2019). A mechanically robust double-network hydrogel with high thermal responses via doping hydroxylated boron nitride nanosheets. J. Mater. Sci..

[98] Gu Z., Huang K., Luo Y., Zhang L., Kuang T., Chen Z., Liao G. (2018). Double network hydrogel for tissue engineering. Wiley Interdiscip. Rev. Nanomed. Nanobiotechnol..

[99] Guiseppi-Elie A. (2010). Electroconductive hydrogels: Synthesis, characterization and biomedical applications. Biomaterials.

[100] Luo Y., Kirker K.R., Prestwich G.D. (2000). Cross-linked hyaluronic acid hydrogel films: New biomaterials for drug delivery. J. Control. Release.

[101] Larrañeta E., Henry M., Irwin N.J., Trotter J., Perminova A.A., Donnelly R.F. (2018). Synthesis and characterization of hyaluronic acid hydrogels crosslinked using a solvent-free process for potential biomedical applications. Carbohydr. Polym..

[102] Chirani N., Yahia L., Gritsch L., Motta F.L., Chirani S., Fare S. (2015). History and Applications of Hydrogels. J. Biomed. Sci..

[103] Kopeček J. (2007). Hydrogel biomaterials: A smart future?. Biomaterials.

[104] Liu M., Zeng X., Ma C., Yi H., Ali Z., Mou X., He N., Li S., Deng Y. (2017). Injectable hydrogels for cartilage and bone tissue engineering. Bone Res..

[105] Rey-Rico A., Madry H., Cucchiarini M. (2016). Hydrogel-based controlled delivery systems for articular cartilage repair. BioMed Res. Int..

[106] Szychlinska M.A., D’Amora U., Ravalli S., Ambrosio L., Di Rosa M., Musumeci G. (2019). Functional Biomolecule Delivery Systems and Bioengineering in Cartilage Regeneration. Curr. Pharm. Biotechnol..

[107] Chai Q., Jiao Y., Yu X. (2017). Hydrogels for biomedical applications: Their characteristics and the mechanisms behind them. Gels.

[108] Liu Y., Hsu S. (2018). Synthesis and biomedical applications of self-healing hydrogels. Front. Chem..

[109] Gaharwar A.K., Avery R.K., Assmann A., Paul A., McKinley G.H., Khademhosseini A., Olsen B.D. (2014). Shear-thinning nanocomposite hydrogels for the treatment of hemorrhage. ACS Nano.

[110] Liu B., Wang Y., Miao Y., Zhang X., Fan Z., Singh G., Zhang X., Xu K., Li B., Hu Z. (2018). Hydrogen bonds autonomously powered gelatin methacrylate hydrogels with super-elasticity, self-heal and underwater self-adhesion for sutureless skin and stomach surgery and E-skin. Biomaterials.

[111] Loebel C., Rodell C.B., Chen M.H., Burdick J.A. (2017). Shear-thinning and self-healing hydrogels as injectable therapeutics and for 3D-printing. Nat. Protoc..

[112] Wang L.L., Highley C.B., Yeh Y.C., Galarraga J.H., Uman S., Burdick J.A. (2018). Three-dimensional extrusion bioprinting of single-and double-network hydrogels containing dynamic covalent crosslinks. J. Biomed. Mater. Res. A.

[113] Rodell C.B., MacArthur J.W., Dorsey S.M., Wade R.J., Wang L.L., Woo Y.J., Burdick J.A. (2015). Shear-thinning supramolecular hydrogels with secondary autonomous covalent crosslinking to modulate viscoelastic properties in vivo. Adv. Funct. Mater..

[114] Gaffey A.C., Chen M.H., Venkataraman C.M., Trubelja A., Rodell C.B., Dinh P.V., Atluri P., Hung G., MacArthur J.W., Soopan R.V. (2015). Injectable shear-thinning hydrogels used to deliver endothelial progenitor cells, enhance cell engraftment, and improve ischemic myocardium. J. Thorac. Cardiovasc. Surg..

[115] Hong S.H., Kim S., Park J.P., Shin M., Kim K., Ryu J.H., Lee H. (2018). Dynamic bonds between boronic acid and alginate: Hydrogels with stretchable, self-healing, stimuli-responsive, remoldable, and adhesive properties. Biomacromolecules.

[116] Dankers P.Y., Hermans T.M., Baughman T.W., Kamikawa Y., Kieltyka R.E., Bastings M.M., Bosman A.W., Janssen H.M., Larsen A., Bosman A.W. (2012). Hierarchical formation of supramolecular transient networks in water: A modular injectable delivery system. Adv. Mater..

[117] Kim S.H., An Y.H., Kim H.D., Kim K., Lee S.H., Yim H.G., Hwang N.S., Kim B.G. (2018). Enzyme-mediated tissue adhesive hydrogels for meniscus repair. Int. J. Biol. Macromol..

[118] Tendulkar G., Chen T., Ehnert S., Kaps H.-P., Nüssler A.K. (2019). Intervertebral Disc Nucleus Repair: Hype or Hope?. Int. J. Mol. Sci..

[119] Growney Kalaf E.A., Flores R., Bledsoe J.G., Sell S.A. (2016). Characterization of slow-gelling alginate hydrogels for intervertebral disc tissue-engineering applications. Mater. Sci. Eng. C.

[120] Bron J.L., Vonk L.A., Smit T.H., Koenderink G.H. (2011). Engineering alginate for intervertebral disc repair. J. Mech. Behav. Biomed. Mater..

[121] Sun Z., Luo B., Liu Z., Huang L., Liu B., Ma T., Luo Z., Gao B., Liu Z.H., Huang J.H. (2016). Effect of perfluorotributylamine enriched alginate on nucleus pulposus cell: Implications for intervertebral disc regeneration. Biomaterials.

[122] Hayami J.W.S., Waldman S.D., Amsden B.G. (2016). Chondrocyte Generation of Cartilage-Like Tissue Following Photoencapsulation in Methacrylated Polysaccharide Solution Blends. Macromol. Biosci..

[123] Karimi Z., Ghorbani M., Hashemibeni B., Bahramian H. (2015). Evaluation of the proliferation and viability rates of nucleus pulposus cells of human intervertebral disk in fabricated chitosan-gelatin scaffolds by freeze-drying and freeze gelation methods. Adv. Biomed. Res..

[124] Zhou X., Tao Y., Wang J., Liu D., Liang C., Li H., Chen Q. (2016). Three-dimensional scaffold of type II collagen promotes the differentiation of adipose-derived stem cells into a nucleus pulposus-like phenotype. J. Biomed. Mater. Res. Part A.

[125] Khang G., Lee S.K., Kim H.N., Silva-Correia J., Gomes M.E., Viegas C.A.A., Reis R.L., Dias I.R., Oliveira J.M. (2015). Biological evaluation of intervertebral disc cells in different formulations of gellan gum-based hydrogels. J. Tissue Eng. Regen. Med..

[126] Ahmad S., Ahmad M., Manzoor K., Purwar R., Ikram S. A review on latest innovations in natural gums-based hydrogels: Preparations & applications. Int. J. Biol. Macromol..

[127] Crevensten G., Walsh A.J., Ananthakrishnan D., Page P., Wahba G.M., Lotz J.C., Berven S. (2004). Intervertebral disc cell therapy for regeneration: Mesenchymal stem cell implantation in rat intervertebral discs. Ann. Biomed. Eng..

[128] Tsaryk R., Silva-Correia J., Oliveira J.M., Unger R.E., Landes C., Brochhausen C., Kirkpatrick C.J., Ghanaati S., Reis R.L. (2017). Biological performance of cell-encapsulated methacrylated gellan gum-based hydrogels for nucleus pulposus regeneration. J. Tissue Eng. Regen. Med..

[129] Van Uden S., Silva-Correia J., Oliveira J.M., Reis R.L. (2017). Current strategies for treatment of intervertebral disc degeneration: Substitution and regeneration possibilities. Biomater. Res..

[130] Li J., Mooney D.J. (2016). Designing hydrogels for controlled drug delivery. Nat. Rev. Mater..

[131] Priya S.G., Jungvid H., Kumar A. (2008). Skin tissue engineering for tissue repair and regeneration. Tissue Eng. Part B Rev..

[132] Pixley S.K., Hopkins T.M., Little K.J., Hom D.B. (2016). Evaluation of peripheral nerve regeneration through biomaterial conduits via micro-CT imaging. Laryngoscope Investig. Otolaryngol..

[133] Longo U.G., Lamberti A., Petrillo S., Maffulli N., Denaro V. (2012). Scaffolds in tendon tissue engineering. Stem Cells Int..

[134] Ramos D., Peach M.S., Mazzocca A.D., Yu X., Kumbar S.G. (2015). Tendon tissue engineering. Regenerative Engineering of Musculoskeletal Tissues and Interfaces.

[135] Kishore V., Alapan Y., Iyer R., Mclay R., Gurkan U.A. (2016). Application of Hydrogels in Ocular Tissue Engineering. GELS Handbook: Fundamentals, Properties and Applications.

[136] Ahearne M. (2014). Development of an ECM hydrogel for corneal tissue engineering. Acta Ophthalmol..

[137] Peppas N.A. (2010). Biomedical Applications of Hydrogels Handbook.

[138] Feksa L.R., Troian E.A., Muller C.D., Viegas F., Machado A.B., Rech V.C. (2018). Hydrogels for biomedical applications. Nanostructures for the Engineering of Cells, Tissues and Organs: From Design to Applications.

[139] Guenther M., Gerlach G., Wallmersperger T., Avula M.N., Cho S.H., Xie X., Scholz C., Devener B.V., Tathireddy P., Magda J.J. (2013). Smart hydrogel-based biochemical microsensor array for medical diagnostics. Advances in Science and Technology.

[140] Zhang Y.S., Khademhosseini A. (2017). Advances in engineering hydrogels. Science.

[141] Leu H.Y., Farhoudi N., Reiche C., Körner J., Mohanty S., Solzbacher F., Magda J. (2018). Low-Cost Microfluidic Sensors with Smart Hydrogel Patterned Arrays Using Electronic Resistive Channel Sensing for Readout. Gels.

[142] Kaiser N.J., Kant R.J., Minor A.J., Coulombe K.L. (2018). Optimizing Blended Collagen-Fibrin Hydrogels for Cardiac Tissue Engineering with Human iPSC-derived Cardiomyocytes. ACS Biomater. Sci. Eng..

[143] Tijore A., Irvine S.A., Sarig U., Mhaisalkar P., Baisane V., Venkatraman S. (2018). Contact guidance for cardiac tissue engineering using 3D bioprinted gelatin patterned hydrogel. Biofabrication.

[144] Boni R., Ali A., Shavandi A., Clarkson A.N. (2018). Current and novel polymeric biomaterials for neural tissue engineering. J. Biomed. Sci..

[145] Woerly S. (1997). Porous hydrogels for neural tissue engineering. Materials Science Forum.

[146] David A., Day J., Shikanov A. (2016). Immunoisolation to prevent tissue graft rejection: Current knowledge and future use. Exp. Biol. Med..

[147] Shrestha P., Regmi S., Jeong J.H. (2019). Injectable hydrogels for islet transplantation: a concise review. J. Pharm. Investig..

[148] Headen D.M. (2017). Microfluidics-Based Microgel Synthesis for Immunoisolation and Immunomodulation in Pancreatic Islet Transplantation. Ph.D. Thesis.

[149] Lu W., Ling M., Jia M., Huang P., Li C., Yan B. (2014). Facile synthesis and characterization of polyethylenimine-coated Fe3O4 superparamagnetic nanoparticles for cancer cell separation. Mol. Med. Rep..

[150] Vatta L.L., Sanderson R.D., Koch K.R. (2006). Magnetic nanoparticles: Properties and potential applications. Pure Appl. Chem..

[151] Cezar C.A., Kennedy S.M., Mehta M., Weaver J.C., Gu L., Vandenburgh H., Mooney D.J. (2014). Biphasic ferrogels for triggered drug and cell delivery. Adv. Healthc. Mater..

[152] Weeber R., Kantorovich S., Holm C. (2015). Ferrogels cross-linked by magnetic particles: Field-driven deformation and elasticity studied using computer simulations. J. Chem. Phys..

[153] Hunt J.A., Chen R., Van Veen T., Bryan N. (2014). Hydrogels for tissue engineering and regenerative medicine. J. Mater. Chem. B.

[154] Wang S., Lee J.M., Yeong W.Y. (2015). Smart hydrogels for 3D bioprinting. Int. J. Bioprinting.

[155] Furth M.E., Atala A., Van Dyke M.E. (2007). Smart biomaterials design for tissue engineering and regenerative medicine. Biomaterials.

[156] Li M., Jilie K. (2007). Smart Hydrogels. Appl. Biotechnol. Biomed..

[157] Ma S., Yu B., Pei X., Zhou F. (2016). Structural hydrogels. Polymer.

[158] Saul J.M., Williams D.F. (2013). Hydrogels in Regenerative Medicine. Handbook of Polymer Applications in Medicine and Medical Devices.

